# Human Semaphorin 3 Variants Link Melanocortin Circuit Development and Energy Balance

**DOI:** 10.1016/j.cell.2018.12.009

**Published:** 2019-02-07

**Authors:** Agatha A. van der Klaauw, Sophie Croizier, Edson Mendes de Oliveira, Lukas K.J. Stadler, Soyoung Park, Youxin Kong, Matthew C. Banton, Panna Tandon, Audrey E. Hendricks, Julia M. Keogh, Susanna E. Riley, Sofia Papadia, Elana Henning, Rebecca Bounds, Elena G. Bochukova, Vanisha Mistry, Stephen O’Rahilly, Richard B. Simerly, INTERVAL, UK10K Consortium, James E.N. Minchin, Inês Barroso, E. Yvonne Jones, Sebastien G. Bouret, I. Sadaf Farooqi

**Affiliations:** 1 University of Cambridge Metabolic Research Laboratories and NIHR Cambridge Biomedical Research Centre, Wellcome-MRC Institute of Metabolic Science, Addenbrooke’s Hospital, Cambridge, UK; 2 The Saban Research Institute, Developmental Neuroscience Program, Center for Endocrinology, Diabetes and Metabolism, Children’s Hospital Los Angeles, University of Southern California, Los Angeles, CA 90027, USA; 3 Center for Integrative Genomics, University of Lausanne, Lausanne, Switzerland; 4 Division of Structural Biology, Wellcome Centre for Human Genetics, University of Oxford, Oxford, UK; 5 Pathogenesis of Vascular Infections Unit, INSERM, Institut Pasteur, Paris, France; 6 School of Biological and Marine Sciences, University of Plymouth, Plymouth, UK; 7 Centre for Cardiovascular Science, The Queen’s Medical Research Institute, University of Edinburgh, UK; 8 Wellcome Sanger Institute, Cambridge, UK; 9 Department of Mathematical and Statistical Sciences, University of Colorado-Denver, Denver, CO 80204, USA; 10 The Blizard Institute, Barts and The London School of Medicine and Dentistry, Queen Mary University of London, London, UK; 11 Department of Molecular Physiology and Biophysics, Vanderbilt University, Nashville, Tennessee, TN 37232-0615, USA; 12 INSERM U1172, Jean-Pierre Aubert Research Center, Lille, France

**Keywords:** Semaphorin 3s, Neuropilins, Plexins, hypothalamus, Pomc, AgRP, obesity

## Abstract

Hypothalamic melanocortin neurons play a pivotal role in weight regulation. Here, we examined the contribution of Semaphorin 3 (SEMA3) signaling to the development of these circuits. In genetic studies, we found 40 rare variants in *SEMA3A-G* and their receptors (*PLXNA1-4; NRP1-2*) in 573 severely obese individuals; variants disrupted secretion and/or signaling through multiple molecular mechanisms. Rare variants in this set of genes were significantly enriched in 982 severely obese cases compared to 4,449 controls. In a zebrafish mutagenesis screen, deletion of 7 genes in this pathway led to increased somatic growth and/or adiposity demonstrating that disruption of Semaphorin 3 signaling perturbs energy homeostasis. In mice, deletion of the Neuropilin-2 receptor in Pro-opiomelanocortin neurons disrupted their projections from the arcuate to the paraventricular nucleus, reduced energy expenditure, and caused weight gain. Cumulatively, these studies demonstrate that SEMA3-mediated signaling drives the development of hypothalamic melanocortin circuits involved in energy homeostasis.

## Highlights

• Rare variants affecting Semaphorin 3 signaling are associated with human obesity
• Disruption of Semaphorin 3 signaling leads to weight gain in zebrafish and mice
• Semaphorin 3 signaling promotes the development of hypothalamic melanocortin circuits

## Graphical Abstract

**Figure:**
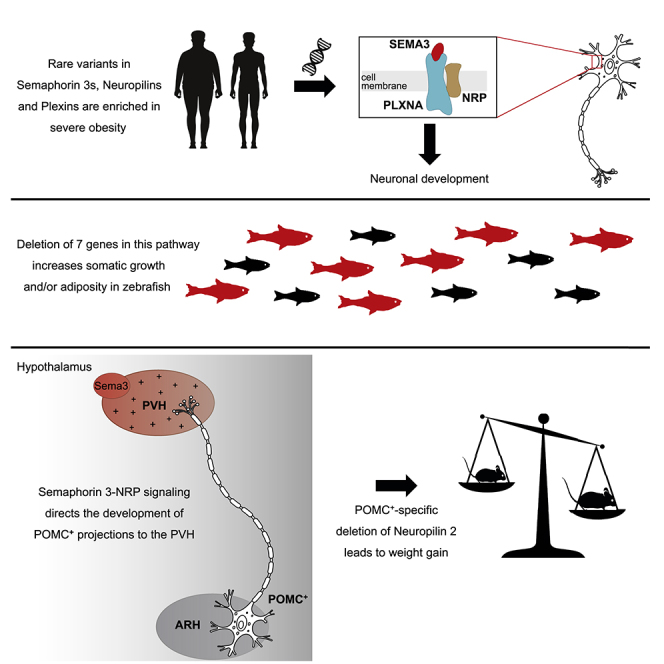

## Teaser

Semaphorin 3 signaling promotes the development of hypothalamic circuits, and human variants are associated with obesity.

## Introduction

Neural circuits in the hypothalamus play a critical role in the regulation of energy homeostasis (Elmquist et al., 1998). The hypothalamic melanocortin circuit is formed by leptin-responsive neurons in the arcuate nucleus of the hypothalamus (ARH) expressing either pro-opiomelanocortin (POMC) or Neuropeptide Y (NPY)/Agouti-related protein (AgRP), which project to, and synapse with, melanocortin-4 receptor (MC4R)-expressing neurons in the paraventricular nucleus of the hypothalamus (PVH). In the nutritionally replete state, ARH POMC neurons release melanocortin peptides, including α-melanocyte-stimulating hormone (α-MSH), which act as agonists at MC4R to reduce food intake and increase energy expenditure (Cowley et al., 2001). Genetic disruption of POMC and MC4R leads to severe obesity in rodents (Huszar et al., 1997, Yaswen et al., 1999) and humans (Krude et al., 1998, Vaisse et al., 1998, Yeo et al., 1998), emphasizing the critical role of this melanocortin circuit in energy homeostasis.

Here, we studied the development of hypothalamic melanocortin circuits, focusing on the contribution of the class 3 Semaphorins (Pasterkamp, 2012) (SEMA3A-G), which direct the development of gonadotropin-releasing hormone (GnRH) neurons into the hypothalamus (Cariboni et al., 2015, Hanchate et al., 2012). Disruption of Sema3 signaling impairs the development of GnRH projections in mice and rare variants that disrupt SEMA3 signaling are associated with hypogonadotropic hypogonadism in humans (Cariboni et al., 2015, Young et al., 2012). We performed genetic studies in people with severe obesity to test whether there was an enrichment of rare potentially functional variants in genes encoding the SEMA3s and their receptors compared to controls. We found 40 rare variants in these genes; 34 altered the function of these proteins through multiple molecular mechanisms. To test whether disruption of Sema3 signaling can perturb energy homeostasis, we performed a CRISPR/Cas9 mutant screen of these genes in zebrafish. We showed that disruption of 7 genes caused increased somatic growth and/or adiposity. Using hypothalamic explants from mice, we demonstrated that Sema3 signaling via Nrp2 receptors drives the development of Pomc projections from the ARH to the PVH. Deletion of Nrp2 in Pomc neurons, reduced the density of Pomc projections and caused weight gain in young mice. Together, these findings demonstrate the role of Sema3 signaling in the development of melanocortin circuits that modulate energy homeostasis, findings that have relevance to the understanding of disorders of human hypothalamic development.

## Results

### Identification of Rare Variants in Semaphorin 3 Ligands and Their Receptors in Severely Obese Individuals

We hypothesized that if the genes encoding Sema3s and their receptors (*SEMA3A-G*, *NRP1*-2, and *PLXNA1-4*) contribute to the development of neurons that regulate body weight in humans, we might identify functional variants in these genes in people with severe early onset obesity. We examined exome sequencing data from 573 individuals with severe early onset obesity (BMI SDS > 3; onset under 10 years of age) recruited to the Genetics of Obesity Study (GOOS) studied as part of the UK10K consortium (Hendricks et al., 2017, Walter et al., 2015). We found 40 rare variants in these 13 genes (Table 1). To test whether there was an enrichment for very rare (minor allele frequency < 0.025%) predicted functional variants in 13 genes involved in Semaphorin 3 signaling in severely obese cases compared to controls, we compared exome sequencing data from the final UK10K data release of 982 severely obese individuals (including the 573 individuals in whom the first 40 variants were identified) with that of 4,449 healthy controls recruited to the INTERVAL study (Moore et al., 2014). After adjusting for multiple testing, we found that very rare predicted functional variants in this cluster of genes were enriched in severely obese cases compared to controls (OR = 1.40, p-adjusted = 0.02; Tables S1 and S2). Although suggestive, these associations were not statistically significant at the single gene level after adjusting for multiple testing. Given the rarity of variants, larger sample sizes will be needed to test whether the burden of rare variants in specific genes or combinations of genes is greater than expected in severely obese cases versus controls.

**Table 1:** Phenotypes of *SEMA3A-G*, *PLXNA1-4*, and *NRP1-2* Variant Carriers

Gene	Variant	Age (years)	BMI (kg/m^2^)	BMI SDS	Endocrine	Neuro developmental	Gut	Other
NRP1; PLXNA3^b^	R237Q^a^; T1679I	2.8	29.3	5.41				
NRP2	V573L	34.1	50.0	-				
NRP2	A506V	3.9	23.8	4.15		autism, speech delay		
NRP2	A506V	11.3	24.1	2		severe migraine		
PLXNA1	L1278F	6.3	28.7	4.53		behavioral problems		
PLXNA1	G1650S	11.9	38.7	3.7				
PLXNA1	R378H	21.0	34.8	2.89		hypotonia, autism, speech delay		
PLXNA2	T515M	14.0	35.9	3.32				
PLXNA2	N788I	6.3	26.0	3.65				
PLXNA2	W25X	11.0	34.2	3.48				
PLXNA2	R1668Q	33.2	63.4	–		Asperger’s syndrome, speech delay		
PLXNA2	A436V	6.2	26.0	3.7				
PLXNA3	V879M	53.7	49.0	–	hypothyroidism		severe constipation	
PLXNA3	R1116C	3.5	23.3	3.71				
PLXNA3	D1710N^a^	13.9	29.4	2.72	hypogonadotropic hypogonadism			
PLXNA3; PLXNA3^b^	D127N; R351H	6.9	26.8	3.59				
PLXNA4	V245G	15.3	58.6	4.63	hypothyroidism	nocturnal enuresis	severe constipation	
PLXNA4	G643D	5.5	27.2	4.19				recurrent infections
PLXNA4	T1642I	11.1	32.8	3.32				
PLXNA4	R70Q	9.5	26.6	3.03		behavioral problems		
SEMA3A	K600M	2.3	29.5	5.5	hypothyroidism			
SEMA3A	R350T	7.7	30.2	3.8	hypothyroidism			
SEMA3B	F355L	10.0	28.4	3.16				
SEMA3B	P296L	13.2	33.4	3.14		epilepsy, narcolepsy, conductive hearing loss, impaired pain sensation, behavioral problems, speech delay		recurrent infections
SEMA3C	R739Q	15.5	43.6	3.82	hypothyroidism	Asperger’s syndrome	severe constipation	
SEMA3D	R773G	11.9	41.3	3.82		nocturnal enuresis		
SEMA3D	R265H	13.0	35.8	3.4				
SEMA3D	T397A	8.9	36.1	4.02				
SEMA3D	Y199S	14.4	37.4	3.44				
SEMA3D	N444S	34.1	41.1	–				
SEMA3D	D380H	16.0	56.4	4.56	hypothyroidism			
SEMA3D	D640Y	7.5	29.2	4.01		autism, learning difficulties, speech delay	severe constipation	
SEMA3E	R167G	14.1	40.5	3.72				
SEMA3E	K711N	7.1	23.1	2.75				
SEMA3F	E88K	2.8	34.8	6.4			severe constipation	
SEMA3G	R561W	3.7	21.3	2.98				recurrent infections
SEMA3G	R728C	13.9	36.1	3.36				
SEMA3G	E478D	15.5	43.8	3.9				
SEMA3G	A86S	14.9	34.6	3.11				

All variants were in found in the heterozygous form unless indicated; gastrointestinal motility disorders presented as severe therapy-resistant constipation. Abbreviations: BMI, body mass index; BMI SDS, BMI standard deviation score for children.

See also Tables S1 and S2.

a Homozygous

b Two participants harbored two variants.

### Rare Variants in *SEMA3*s Affect Their Secretion and Function in Cells

**Figure 1:**
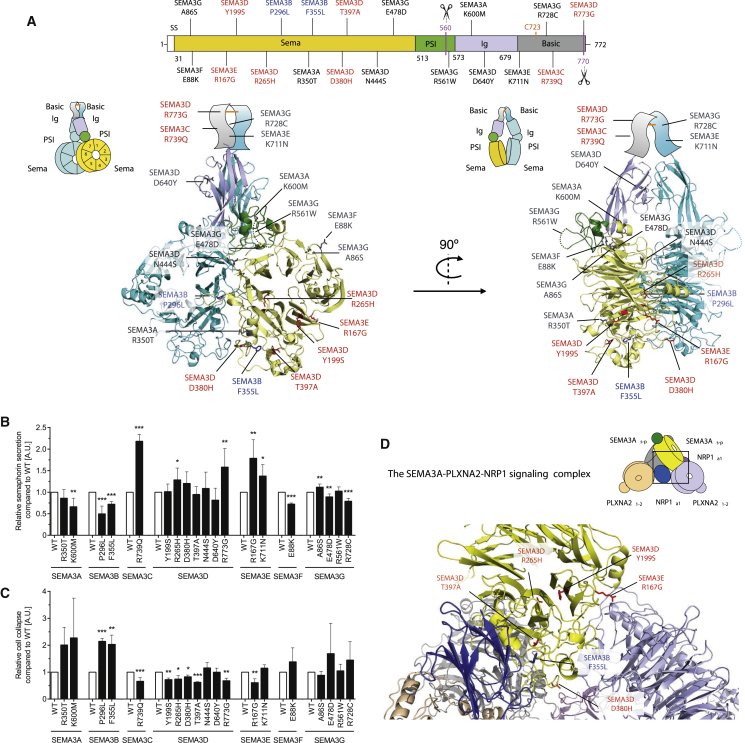
Rare Human Variants in *SEMA3A-G* Disrupt Protein Secretion and Signaling (A) Structural modeling of *SEMA3* variants. Upper panel: *SEMA3* variants on a schematic representation (mouse Sema3A numbering). SS, signal sequence; Sema, semaphorin domain; PSI, plexin-semaphorin-integrin domain; conserved furin cleavage sites indicated by scissors; conserved cysteines that form SEMA3A-G dimers (orange line). Lower panel: *SEMA3A-G* mutants mapped onto human SEMA3A structure (increase, blue; decrease, red; no effect, gray; on U87MG cell collapse). Sema and PSI domains on mouse Sema3A crystal structure (PDB: 4GZ8); Ig domain, model combining human SEMA4D (PDB: 1OLZ) and mouse Sema3A (PDB: 4GZ8) structural data; c-terminal basic domain, schematic. (B) ELISA analysis of C-FLAG-tagged WT/mutant SEMA3A-G secreted in the medium (a.u., arbitrary units). (C) Effect of WT/mutant SEMA3A-G on cell collapse normalized to amount of semaphorin secreted. (D) Structural analysis of *SEMA3* mutants affecting cell collapse (increased, blue; decreased, red). Mutants are mapped on the crystal structure of the mouse Sema3A-Nrp1-PlxnA2 complex (PDB: 4GZA). Data represented as mean ± SEM from at least three independent experiments. ^∗^p < 0.05; ^∗∗^p < 0.01; ^∗∗∗^p < 0.001 for all experiments. See also Figure S1 and Table S3.

**Figure S1:**
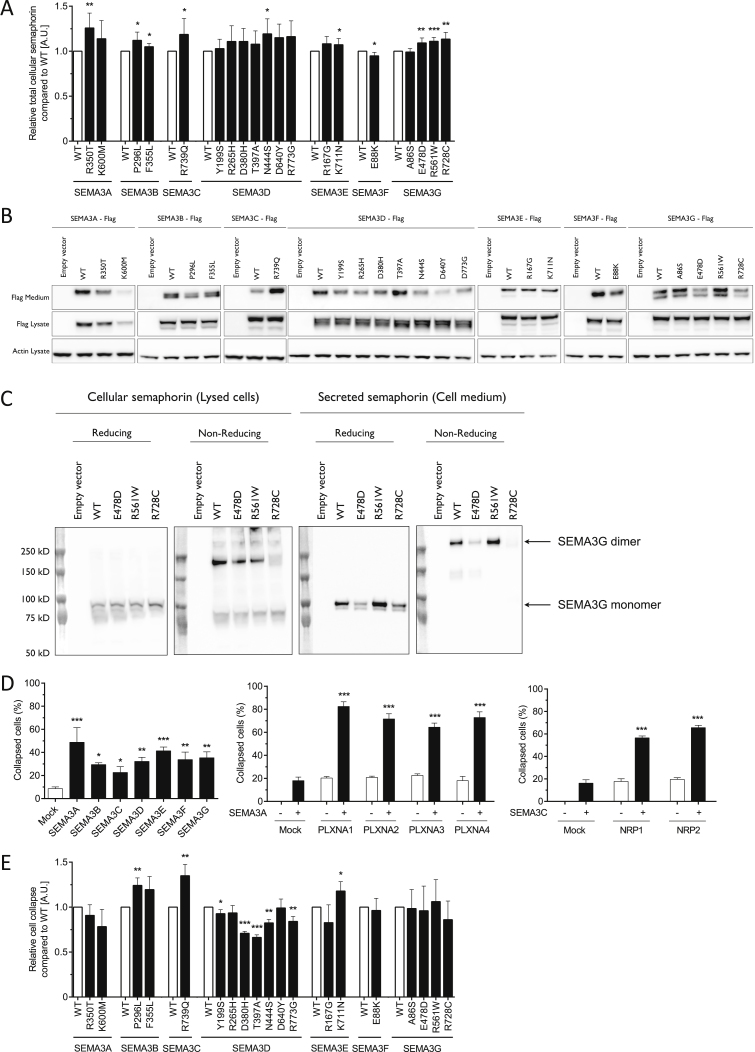
Functional Characterization of Rare Human Variants in SEMA3A-G, Related to Figure 1 (A) Total expression of C-FLAG-tagged SEMA3A-G by ELISA analysis (A.U., arbitrary units). (B) Western blotting of total cellular and secreted SEMA3A-G. (C) Dimerization analysis using reducing and non-reducing western blotting of total cellular and secreted SEMA3G. (D) Collapse efficiency was assessed by counting the proportion of collapsed cells 30 min following addition of the indicated WT Semaphorin to the culture medium. (E) Effect of SEMA3A-G on cell collapse unadjusted for the amount of semaphorin secreted. Data are presented as mean ± SEM from at least 3 independent experiments; ^∗^p < 0.05, ^∗∗^p < 0.01 and ^∗∗∗^p < 0.001.

We performed experiments in cells to dissect the functional consequences of very rare human variants (Figures 1 and S1; Table S3). SEMA3A-G are secreted as disulphide bridge-linked dimers and processed by furin (Figure 1A). SEMA3s (except SEMA3E) bind to Neuropilin co-receptors (NRP1 and NRP2) in hetero-complexes with PlexinA1-4 (PLXNA1-4) receptors to activate plexin signal-transduction (SEMA3E can signal without NRPs through the class D plexin, PLXND1) (Janssen et al., 2012, Tran et al., 2007). The dimeric SEMA3s form the signaling complex with two PLXNAs and NRPs, the NRPs cross-bracing the interfacing SEMA3s and PLXNAs (Janssen et al., 2012).

We mapped the 19 variants in *SEMA3s* onto the crystal structure of SEMA3A and homology models of SEMA3B-3G to suggest structural explanations for our findings (Figure 1A). To assess whether SEMA3s mutants affect protein secretion, we quantified the amount of secreted SEMA3 detected in the medium of HEK293 cells transiently transfected with Flag-tagged wild-type (WT) or mutant SEMA3 by ELISA. Six mutants decreased protein secretion compared to WT SEMA3 (Figure 1B). Most led to increased intracellular retention of mutant SEMA3, suggesting that the defect was in secretion rather than synthesis (Figure S1A). In contrast, six mutants led to increased protein secretion (Figures 1B and S1B). The *SEMA3G* R728C variant may hinder SEMA3 dimerization by disrupting the formation of an intersubunit disulfide bridge by the proximal, conserved cysteine residue C726 (Figures 1A and S1C).

To test whether SEMA3 mutants affect receptor-mediated signaling and thus disassembly of the actin cytoskeleton and cellular collapse, U97MG cells were treated with medium from cells transfected with WT or mutant SEMA3s, and the number of collapsed cells counted. Compared to WT SEMA3s, 9 of the 19 SEMA3 mutants affected cell collapse (Figure 1C; Table S3). Five SEMA3D mutants induced less collapse than WT (Figure 1C).

Based on homology modeling, 12 of 19 variants were predicted to affect secretion and/or cellular collapse due to destabilization of the Sema domain important for SEMA3-PLXNA-NRP recognition (Figure 1D). Paradoxically, four mutants decreased collapse despite increased secretion. SEMA3C R739Q and SEMA3D R265H both locate close to the SEMA3-NRP interface and may thus weaken SEMA3C-NRP1/2 binding. SEMA3D R773G may destabilize the SEMA3-PLXNA-NRP complex by affecting the charge distribution on the basic tail. SEMA3E R167G, located at the SEMA3-PLXNA interface, may directly affect PLXN binding (Figure 1D). Two SEMA3B mutants showed decreased secretion, yet increased collapse even after adjustment for the amount of protein secreted (Figures 1B, 1C, S1D, and S1E). In summary, 15 of the 19 variants have functional consequences on the protein by affecting secretion and/or collapse in these assays (Table S3).

### Rare Variants in *NRP1-2* and *PLXNA1-4* Disrupt Cell-Surface Localization and Function

**Figure 2:**
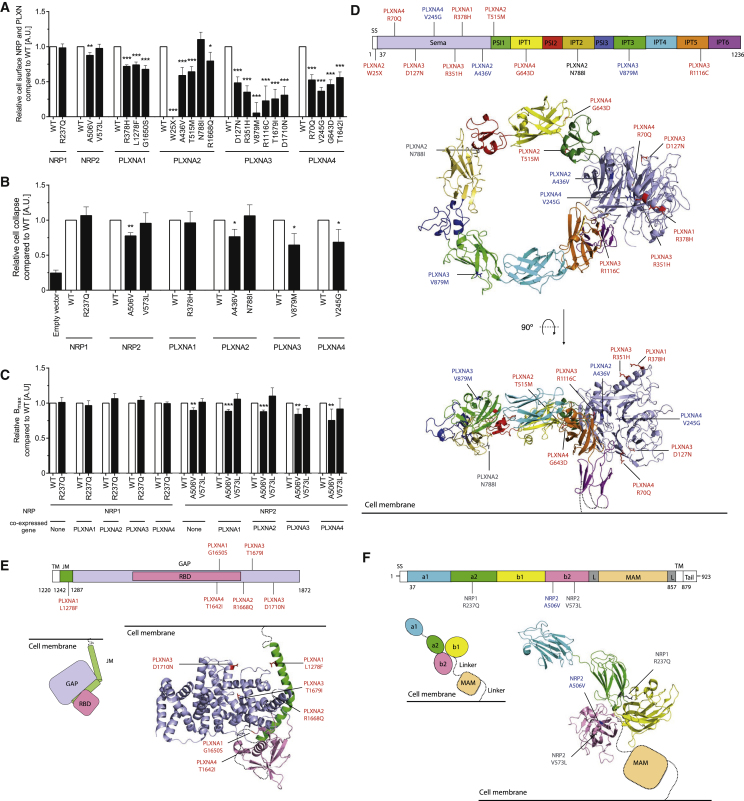
Rare Human Variants in *Neuropilins 1-2 and Plexins A1-4* Disrupt Cellular Localization and Signaling (A) Cell-surface localization for WT/mutant NRP1-2 and PLXNA1-4 by ELISA. (B) Effect of WT/mutant NRP1-2 and PLXNA1-4 on semaphorin-induced cell collapse. (C) Total binding (Bmax) in cells expressing WT/mutant NRP1-2 or co-expressing NRP1-2 mutants and WT PLXNA1-4. (D) Structural modeling of PLXNA mutants. Upper panel: PLXNA1-4 variants shown on schematic (mouse PlxnA1 numbering). SS, signal sequence; PSI, plexin-semaphorin-integrin; IPT, Ig domain. Lower panel: PLXNA mutants mapped onto the crystal structure of mouse PlxnA1 ectodomain (PDB: 5L56). (E) PLXNA mutants mapped onto the crystal structure of mouse PlxnA3 intracellular domain (PDB: 3IG3). (F) Upper panel: NRP variants shown on the schematic (mouse Nrp1 numbering). Lower panel: NRP1-2 variants mapped onto the crystal structure of mouse Nrp1 (PDB: 4GZ9). The membrane-proximal MAM (meprin, A-5 protein, and receptor protein-tyrosine phosphatase mu) domain of Nrp is represented schematically. RBD, Rho GTPase-binding domain; GAP, GTPase-activating protein; TM, transmembrane; JM, Juxtamembrane. The neuropilin ectodomain comprises two CUB domains (a1 and a2), two coagulation factor V/VIII homology domains (b1 and b2) and a MAM domain, L, linker. In (D)–(F), variants causing decreased surface expression (red), no effect on surface expression (gray), decreased surface expression as well as decreased cell collapse (blue) are shown. Data represented as mean ± SEM from at least three independent experiments. ^∗^p < 0.05; ^∗∗^p < 0.01; ^∗∗∗^p < 0.001. See also Figure S2.

**Figure S2:**
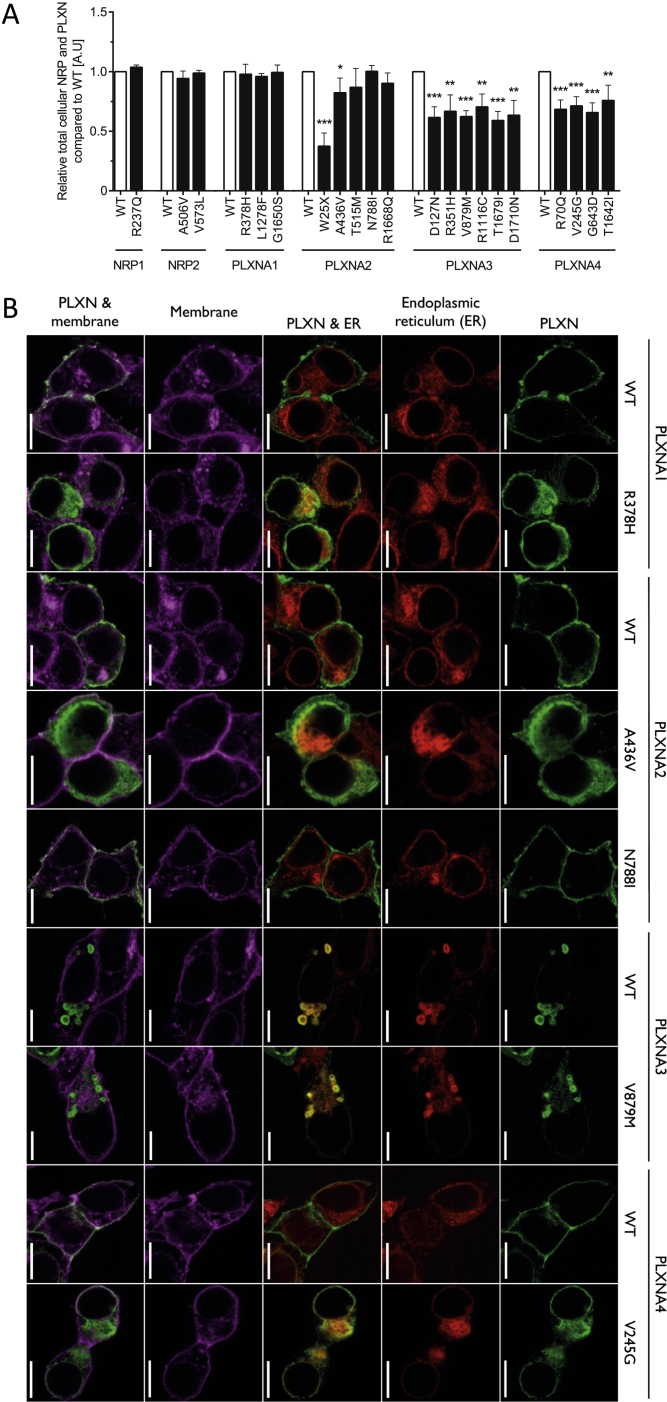

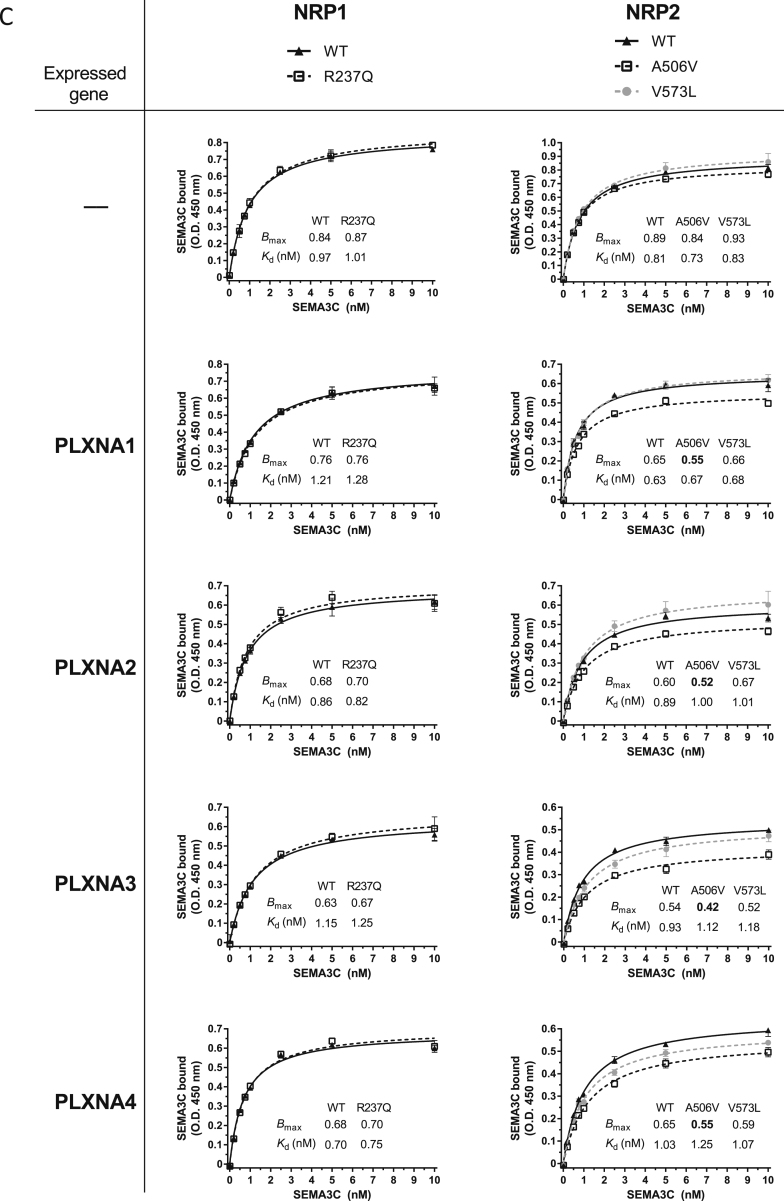

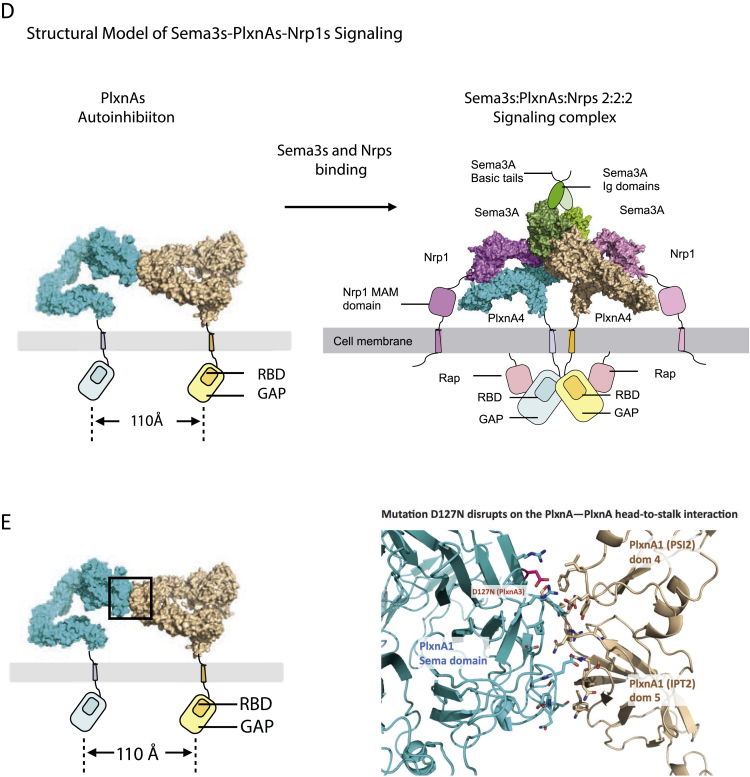
Functional Characterization of Rare Human Variants in Neuropilins 1-2 and Plexins A1-4, Related to Figure 2 (A) Total expression of WT and mutant NRP1-2 and PLXNA1-4 by ELISA on permeabilised HEK293 cells (A.U, arbitrary units). (B) Confocal microscopy of Cos-7 cells showing the co-localization of transiently expressed WT and mutant PLXNA1-4 (green) with plasma membrane (magenta) and endoplasmic reticulum (red) markers. Scale bars represent 10 μm. (C) Saturation receptor-ligand binding assay. Cells expressing WT/mutant NRP1-2 or co-expressing mutant NRP1-2 and WT PLXNA1-4 were incubated with increasing amounts of recombinant human SEMA3C and the equilibrium dissociation constant (kd) of the interaction and the total binding (Bmax) were calculated. (D) Structural model of SEMA3s signaling via the PLXNA receptors and co-receptor NRP1 or 2. (E) Variant *PXLNA2* D127N locates on the PLXNA-PLXNA interface important for pre-signaling auto-inhibition. Data are presented as mean ± SEM from at least 3 independent experiments; ^∗^p < 0.05, ^∗∗^p < 0.01 and ^∗∗∗^p < 0.001.

We examined the molecular mechanisms by which the 21 variants in *PLXNA1-4* and *NRP1-2* might affect their function (Figures 2 and S2). HEK293 cells were transfected with N-terminally GLU-GLU-tagged WT and mutant constructs. Surface localization of NRPs and PLXNs on non-permeabilized cells was quantified by ELISA using an anti-GLU-GLU antibody. One NRP2 mutant (A506V) and 17 of the 18 PLXNA mutants significantly decreased cell-surface expression compared to WT receptors (Figures 2A and S2A). WT PLXNA1, A2, and A4 were predominantly localized on the plasma membrane, whereas mutant PLXNs with reduced cell-surface expression were predominantly found within the endoplasmic reticulum (ER) (Figure S2B). Interestingly, both WT and mutant PLXNA3 were localized in the ER (Figure S2B). Almost all mutants with decreased cell-surface localization reduced cell collapse in cells transfected with NRP or PLXN (Figure 2B). In a ligand binding assay, none of the mutants affected the equilibrium dissociation constant of the interaction (Figure S2C); only NRP2 A506V decreased total binding (Figure 2C), in agreement with its modest decrease in cell-surface expression. Co-expression of NRP mutants with each WT PLXN gave similar results as with expression of NRP alone (Figures 2C and S2C).

Structural modeling of the 21 mutants in PLXNAs and NRPs (Figures 2D–2F) suggested possible explanations for protein misfolding. Six of the 11 mutants lie in the Sema domain of the PlxnA ectodomains; however, they are not at the Sema3 and Nrp-binding surfaces, so are likely to affect structural stability rather than ligand or co-receptor interactions (Figure 2D). Mutants on the PSI and IPT domains may disrupt the stability of these domains (Figure 2D). PLXNA3 D127N is located on a surface that mediates PlxnA-PlxnA interactions important for receptor auto-inhibition pre-ligand binding (Kong et al., 2016) (Figures S2D and S2E). Six mutants lie in the cytoplasmic Plxn domains and may additionally affect coupling to downstream signaling molecules; PLXNA1 L1278F on the juxta-membrane helices may affect pre-signaling inhibition, PLXNA3 D1710N on the Rap-GAP pocket helices may affect Rap binding, PLXNA1 G1650S, and PLXNA4 T1642I on the Rho-GTPase binding domain (RBD) may affect interactions with the Rho-GTPases and PLXNA3 T1679I may disrupt the hydrophobic core of the PlxnA GAP domain (Figure 2E). Only 1 of the 3 mutants found on NRP1 and NRP2 lead to decreased cell collapse and reduced cell-surface expression likely by affecting molecular stability (Figure 2F).

### A Mutagenesis Screen in Zebrafish Demonstrates that Disruption of *Semaphorins, Plexins*, and *Neuropilins* Alters Somatic Growth and Adiposity

**Figure 3:**
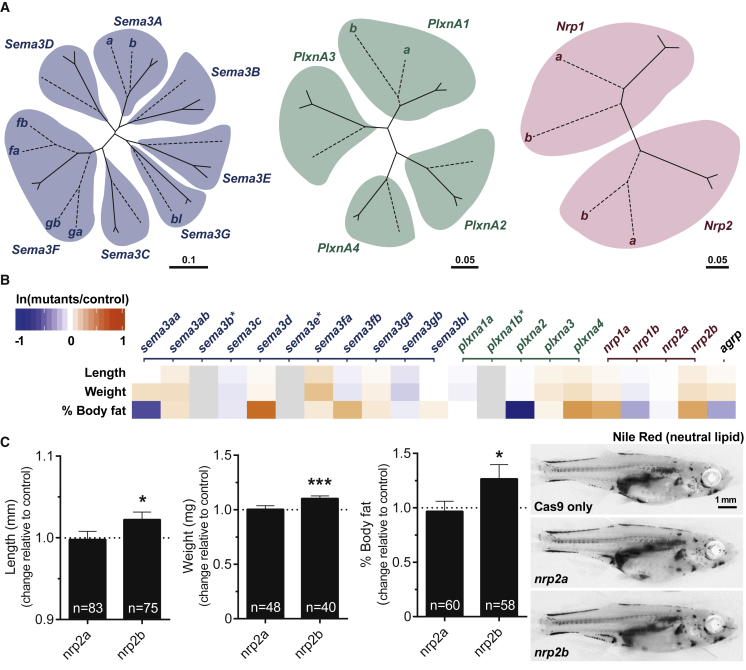
Disruption of *Semaphorin 3 S, Neuropilins*, and *Plexins* Alters Energy Homeostasis in Zebrafish (A) Unrooted phylogenetic trees of Sema3, PlxnA, and Nrp genes. Genes from zebrafish (dotted lines) and mouse and human (solid lines) were used to construct the trees. Where zebrafish genes have been duplicated, a letter is used to identify paralogs. Scale bars, number of substitutions per amino acid site. (B) Heatmap showing change in length, weight, and percentage of body fat in deletion mutants relative to Cas9-only control fish; decrease (blue), increase (orange) in the phenotype of mutants relative to control fish (for the natural log fold change); ^∗^genes not screened; *agrp*, positive control. (C) Length (mm), weight (mg) and percentage of body fat in *nrp2a* and *nrp2b* mutant fish relative to Cas9-only injected control fish. Data represented as mean ± SEM. ^∗^p < 0.05; ^∗∗∗^p < 0.001 in one-sample t tests. Representative images of Nile Red-stained zebrafish showing increased adiposity and size of *nrp2b* mutant fish (right). Scale bar, 1 mm. See also Figure S3.

**Figure S3:**
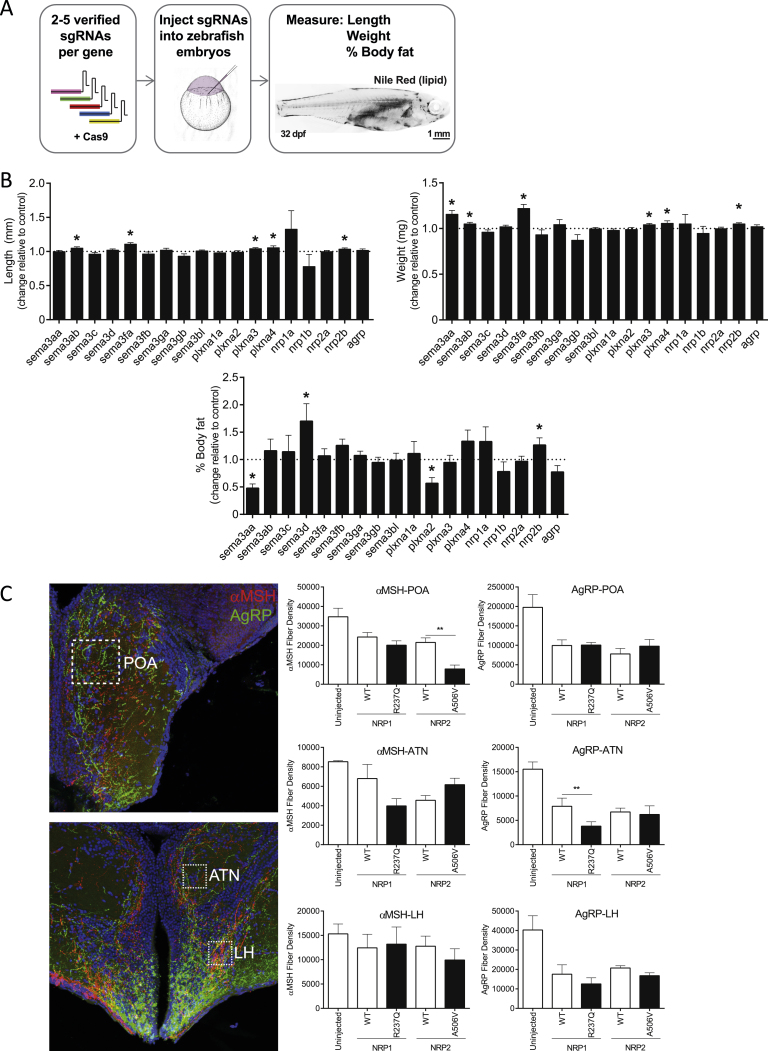
Generation and Characterization of Semaphorin-Neuropilin-Plexin Deletion Mutants in Zebrafish, Related to Figure 3 (A) Schematic illustrating the mutagenesis strategy to target *sema3*, *plxna* and *nrp* genes in zebrafish. Two-five sgRNAs were generated to mutagenize each zebrafish gene. Only sgRNAs verified to induce mutagenesis were injected into one-cell stage zebrafish embryos. Zebrafish were raised to ∼30 days post fertilization and fish length (mm), weight (mg) and % body fat were quantified. (B) Results on fish length (mm), weight (mg) and % body fat for all deletion mutants (summarized in Figure 3B). (C) Microphotographs and quantification of the density of α-melanocyte-stimulating hormone (αMSH) (red) and agouti-related peptide (AgRP) (green) immunoreactive (IR) fibers innervating the preoptic area (POA), anterior tuberal nucleus of hypothalamus (ATN), and lateral hypothalamic nucleus (LH) of 35-day-old wild-type zebrafish overexpressing NRP1 and NRP2; ^∗^p < 0.05 in one-sample t tests.

We used zebrafish to test whether altered Sema3 signaling can disturb energy homeostasis, as the hypothalamic neural circuits involved in energy homeostasis are highly conserved (Leibold and Hammerschmidt, 2015, Minchin and Rawls, 2017). Multiple CRISPR guide RNAs (gRNAs) targeting distinct regions of each zebrafish *semaphorin 3*, *neuropilin*, and *plexin a* ortholog (Figures 3A and S3A) were injected into one-cell stage zebrafish embryos. Disruption of the melanocortin system can influence both somatic growth and adiposity in zebrafish (Sebag et al., 2013, Zhang et al., 2012). Deletions in seven genes significantly increased somatic growth, body weight and/or the percentage of body fat (Figures 3B and S3B). Deletion of a duplicate gene for NRP2 (*nrp2b)* increased adiposity and somatic growth (Figure 3C). In contrast, deletions of two genes decreased percentage of body fat. Cumulatively, these data demonstrate that Semaphorin 3 signaling can affect energy homeostasis, potentially through several different mechanisms.

### SEMA3s and Their Receptors Are Expressed in the Hypothalamus during Early Development

**Figure 4:**
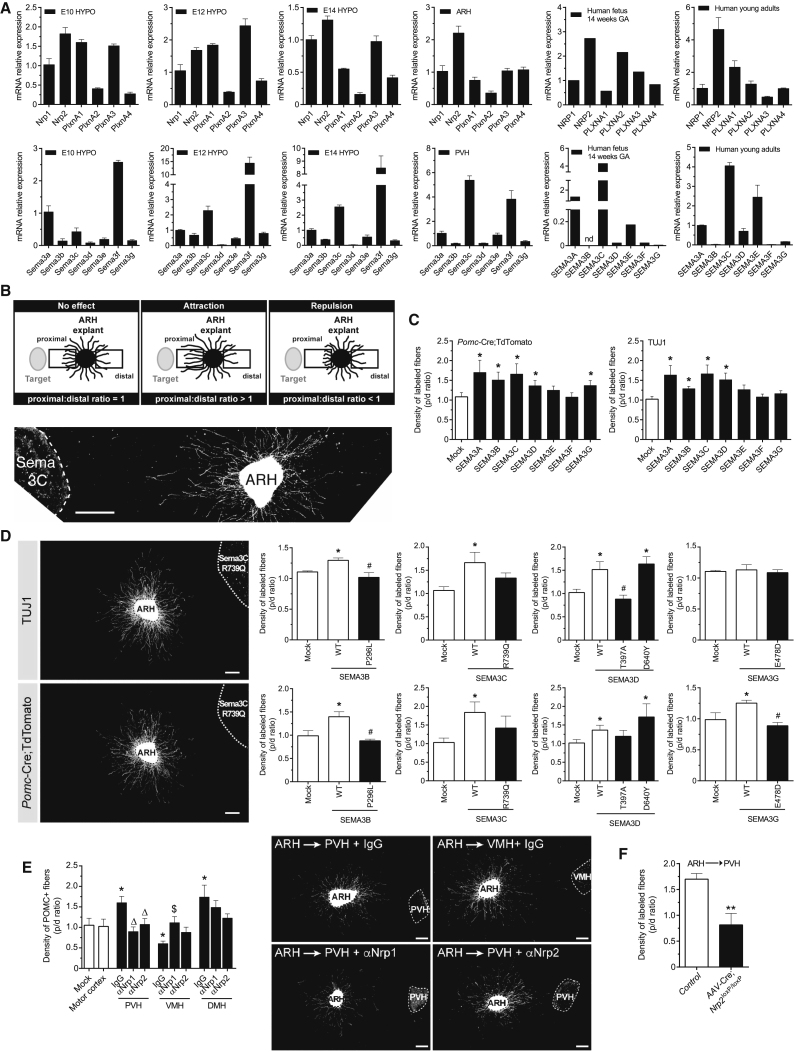
Class 3 Semaphorins and their receptors are expressed in the developing hypothalamus and direct innervation of the paraventricular nucleus of the hypothalamus by arcuate Pomc axons (A) Expression of Neuropilin (*NRP1-2*), PlexinA (*PLXNA1-A4*), and Semaphorin (*SEMA3A-G*) mRNA in the hypothalamus (HYPO) of mouse fetuses at embryonic day E10/12/14, in hypothalamic nuclei of P10 mice (ARH-arcuate; PVH-paraventricular nucleus of the hypothalamus), in the hypothalamus from human fetuses at 14 weeks of gestational age (GA), and from human young adults; values relative to GAPDH expression shown. (B) In a co-culture system to evaluate neural growth, the average density of neurites in the proximal and distal parts of the ARH explant (with respect to the target tissue, e.g., PVH) is compared to quantify the density of axons extending toward (proximal) or away (distal) from cell aggregates. (C) ARH explants from *Pomc*-Cre; TdTomato mice were co-cultured with an aggregate of HEK293 cells overexpressing Sema3A-G and immunostained with TUJ1 (neuron-specific class III beta-tubulin). (D) Quantitative analysis of TUJ1^+^ (upper panel) and Pomc^+^ (lower panel) axons derived from arcuate explants co-cultured with an aggregate of HEK293 cells overexpressing *SEMA3A-G* mutants (^∗^p < 0.05 versus mock; ^#^p < 0.05 versus WT). ARH, arcuate nucleus. (E) ARH explants derived from *Pomc*-Cre, TdTomato mice were co-cultured with explants containing the PVH, DMH, or VMH and Nrp1 or Nrp2 blocking antibodies (α). Data represented as mean ± SEM. ^∗^p < 0.05 versus mock; Δ, p < 0.05 versus PVH immunoglobin (IgG); $, p < 0.05 versus VMH IgG. (F) ARH explants derived from *Nrp2*^loxP/loxP^ mice that received intra-ARH injections of an AAV-Cre vector with explants containing the PVH. Genetic loss of Nrp2 in the ARH causes a significant reduction in ARH axon growth. ^∗∗^p < 0.01 versus control. Scale bars, 250 μm (B) and 100 μm (D and E). See also Figure S4.

**Figure S4:**
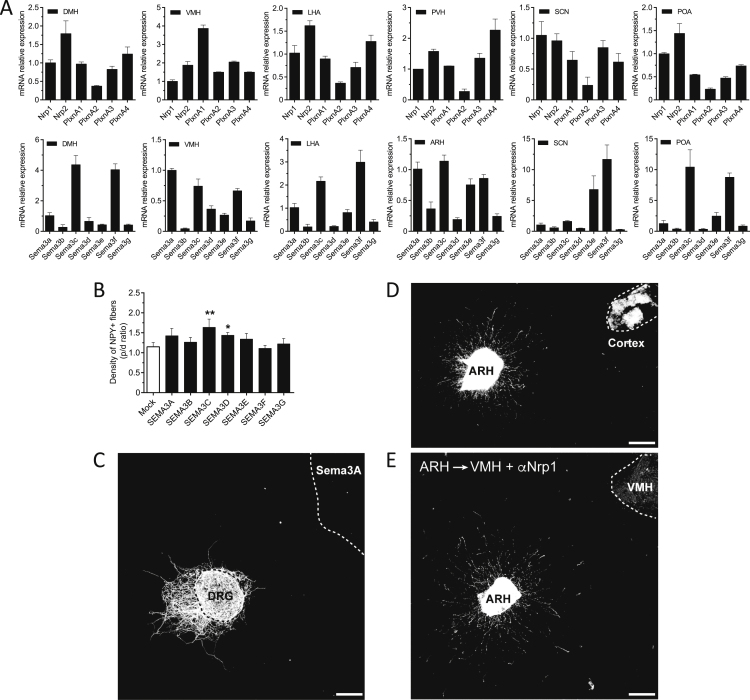
Expression of Class 3 Semaphorins and Their Receptors in the Developing Hypothalamus and Specificity of Co-Culture Assays, Related to Figure 4 (A) Expression of Semaphorin (*Sema3A-G*), Neuropilin (*Nrp1-2*), PlexinA (*PlxnA1-A4*) mRNA in microdissected hypothalamic nuclei of P10 mice; compared to expression of GAPDH. (B) Quantitative analysis of NPY^+^ axons derived from arcuate explants co-cultured with an aggregate of HEK293 cells overexpressing Sema3A-G. (C) Representative image showing an isolated explant derived from the dorsal root ganglion (DRG) and co-cultured with an aggregate of HEK293 cells overexpressing Sema3A. (D) Representative image showing an ARH explant co-cultured with a cortical explant. (E) Representative image showing ARH explants derived from *Pomc*-Cre, TdTomato mice co-cultured with explants containing the VMH in the presence of Nrp1 blocking antibodies (α). Data are represented as mean ± SEM. ^∗^p < 0.05 and ^∗∗^p < 0.01 versus mock. ARH, arcuate nucleus of the hypothalamus; DMH, dorsomedial nucleus of the hypothalamus; LHA, lateral hypothalamic area; POA, preoptic area; SCN, suprachiasmatic nucleus; VMH, ventromedial nucleus of the hypothalamus.

Using qRT-PCR we found that *Nrp1-2* and *PlxnA1-4* mRNAs were detected in the mouse hypothalamus as early as E10 and were highly expressed postnatally, particularly in the ARH (Figures 4A and S4A). *NRP1-2* and *PLXNA1-4* mRNAs were also expressed in the human fetal hypothalamus and the hypothalamus of human young adults (Figure 4A). *Sema3a*, *Sema3c*, *Sema3e*, and *Sema3f* were expressed in the mouse PVH at P10 (Figure 4A); *SEMA3C* was the most abundant in the mouse PVH, DMH, ARH, and preoptic area (Figure S4A) and in the human fetal and young adult hypothalamus (Figure 4A). This temporal pattern of gene expression overlaps with a critical time window for the development of the melanocortin circuits that regulate energy homeostasis. Pomc neurons in the ARH are generated on embryonic day (E)11-12, acquire their terminal peptidergic phenotype during mid-late gestation (Padilla et al., 2010) and send axonal projections to their target sites during the first few weeks of postnatal life (Bouret et al., 2004).

### Sema3s Drive the Growth of Pomc Projections *In Vitro*

To test whether Sema3s are involved in the development of arcuate Pomc projections, we performed co-cultures between ARH explants derived from *Pomc*-Cre; TdTomato mice (to genetically label Pomc axons) and HEK293 cell aggregates transfected with human *SEMA3*-encoding vectors (Figure 4B). We quantified the density of axons extending toward (proximal) or away (distal) from cell aggregates (Figure 4B). Compared with the radial growth seen with HEK293 cells transfected with an empty vector, growth of arcuate Pomc axons was enhanced by HEK293 cell aggregates overexpressing SEMA3A, SEMA3B, SEMA3C, SEMA3D, and SEMA3G (Figure 4C). Notably, only SEMA3C and SEMA3D affected the growth of arcuate NPY axons (Figure S4B). To validate the specificity of our *in vitro* assay, we co-cultured an explant derived from the dorsal root ganglion (DRG) with HEK293 cells overexpressing SEMA3A and confirmed, as previously described (Taniguchi et al., 1997) that SEMA3A inhibited the growth of DRG axons (Figure S4C). Together, these observations demonstrate that SEMA3s can signal to ARH axons, including to Pomc axons, and display enhanced growth.

We repeated co-cultures between mouse ARH explants and HEK293 cell aggregates transfected with a subset of human *SEMA3* variants and evaluated axon growth. Three of five variants tested negatively affected the overall growth of arcuate axons; one increased the density of POMC projections (Figure 4D). Interestingly, *SEMA3G* E478D and *SEMA3B* P296L mutants specifically reduced POMC axon growth.

### Neuropilin Receptors Mediate the Growth of Arcuate Pomc Projections toward the PVH and away from the VMH

We reconstructed arcuate connections *in vitro* by co-culturing two explants derived from different hypothalamic nuclei. When an ARH explant derived from *Pomc*-Cre; TdTomato mice was co-cultured with a PVH explant, the density of fibers directed toward the PVH was substantially greater than that from the opposite side of the ARH, suggesting that the PVH releases diffusible chemotropic factors that promote growth of arcuate Pomc axons (Figure 4E). Substitution of the PVH explants with control HEK293 cells or an explant derived from the cortical cortex (normally not innervated by ARH axons) did not result in any detectable effect on neural projections (Figures 4E and S4D), demonstrating a high degree of specificity. Nrp1 or Nrp2 neutralizing antibodies blocked the induction of growth exerted by the PVH on arcuate Pomc axons (Figure 4E). The DMH promoted growth of arcuate Pomc axons, but this effect was not blocked with Nrp1- or Nrp2-neutralizing antibodies (Figure 4E). In contrast, the VMH inhibited growth of arcuate Pomc axons and this effect was blocked by the addition of Nrp1, but not Nrp2, antibodies (Figure 4E and S4E). Thus, Nrp-mediated signaling plays a specific role in establishing Pomc axonal projections to the PVH.

### Genetic Deletion of Neuropilin 2 Reduces the Density of Pomc Projections to the PVH and Causes Weight Gain in Mice

**Figure S5:**
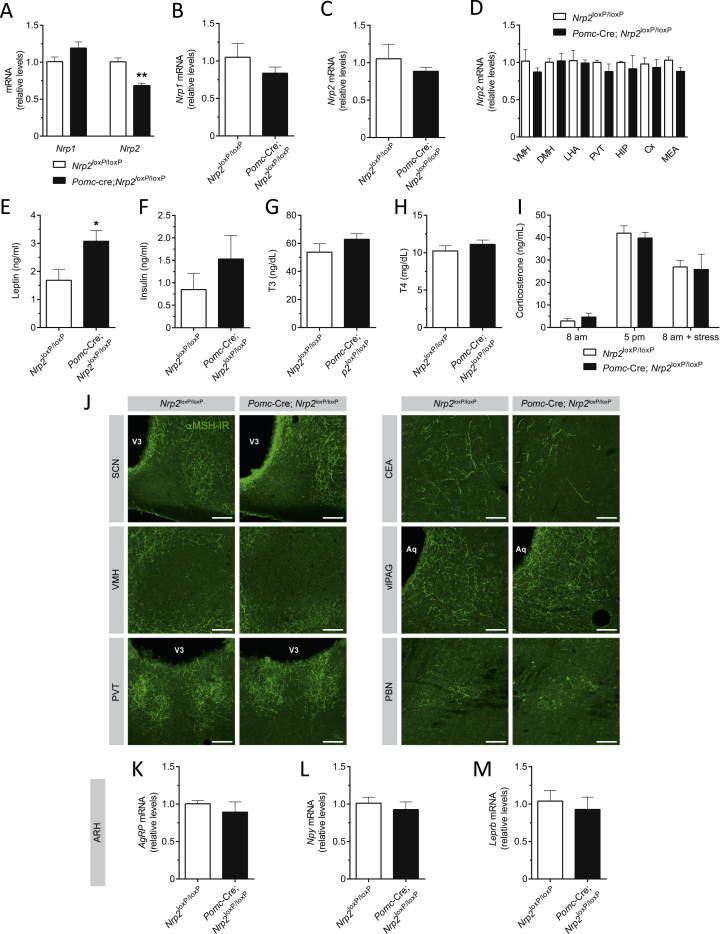
Metabolic and Neuroanatomical Characterization of *Pomc*-Cre; *Nrp2*^loxP/loxP^ Mice, Related to Figure 5 (A) Expression of *Nrp1* and *Nrp2* mRNA in the arcuate nucleus of the hypothalamus of adult *Nrp2*^loxP/loxP^ and *Pomc*-Cre; *Nrp2*^loxP/loxP^ mice; values relative to GAPDH expression are shown. (B–D) (B)Relative levels of *Nrp1* in the pituitary of adult *Nrp2*^loxP/loxP^ (control) and *Pomc*-Cre; *Nrp2*^loxP/loxP^ (mutant) mice. *Nrp2* mRNA expression in (C) the pituitary, and (D) ventromedial nucleus of the hypothalamus (VMH), dorsomedial nucleus of the hypothalamus (DMH), lateral hypothalamic area (LHA), paraventricular nucleus of the thalamus (PVT), hippocampus (HIP), cortex (Cx), and medial amygdala (MEA) of *Nrp2*^loxP/loxP^ (control) and *Pomc*-Cre; *Nrp2*^loxP/loxP^ (mutant) mice. (E–I) (E) Serum leptin, (F) insulin, (G) triiodothyronine (T3), (H) thyroxine (T4), and (I) corticosterone levels of adult *Nrp2*^loxP/loxP^ and *Pomc*-Cre; *Nrp2*^loxP/loxP^ mice. (J) Representative confocal images showing α-melanocyte-stimulating hormone (αMSH)-immunoreactive (IR) fibers in the brain of adult *Nrp2*^loxP/loxP^ and *Pomc*-Cre; *Nrp2*^loxP/loxP^ mice. (K–M) Relative levels of (K) Agouti-related peptide (*Agrp*), (L) neuropeptide Y (*Npy*), and (M) leptin receptor (*Leprb*) mRNA in the arcuate nucleus of the hypothalamus of adult *Nrp2*^loxP/loxP^ and *Pomc*-Cre; *Nrp2*^loxP/loxP^ mice. Data are presented as mean ± SEM. ^∗^p < 0.05, ^∗∗^p < 0.01 versus *Nrp2*^loxP/loxP^ mice. Aq, aqueduct; ARH, arcuate nucleus of the hypothalamus; CEA, central nucleus of the amygdala; vlPAG, ventrolateral periaqueductal gray matter; PBN, parabrachial nucleus; PVT, paraventricular nucleus of the thalamus; SCN, suprachiasmatic nucleus; VMH, ventromedial nucleus of the hypothalamus; V3, third ventricle. Scale bars, 100 μm.

**Figure 5:**
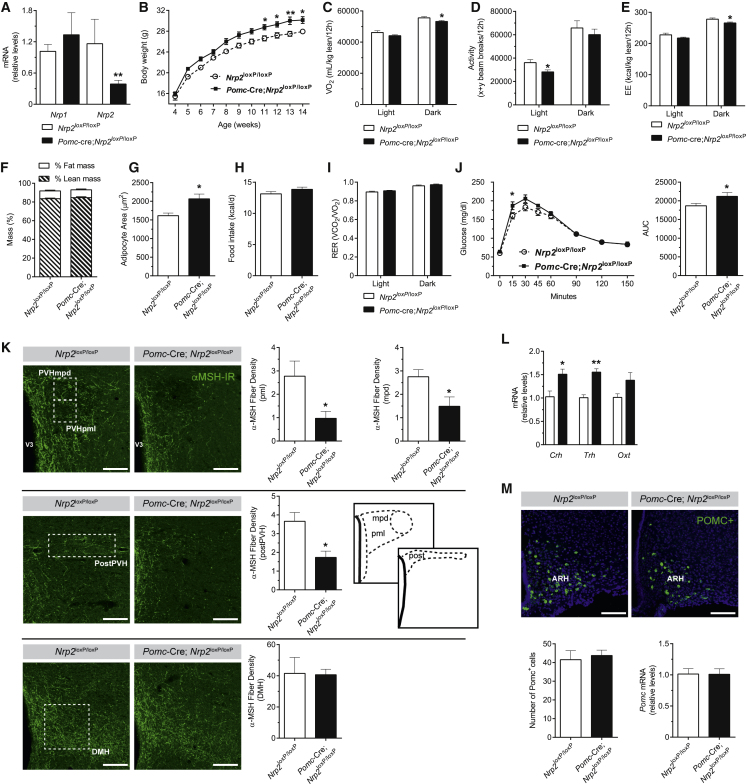
Loss of Neuropilin 2 Signaling in Pomc Neurons Causes Reduced Energy Expenditure and Weight Gain in Mice and Disrupts Arcuate Pomc Projections to the PVH (A) Expression of *Nrp1* and *Nrp2* mRNA in sorted POMC^+^ neurons of adult *Nrp2*^loxP/loxP^ and *Pomc*-Cre; *Nrp2*^loxP/loxP^ mice; values relative to GAPDH expression shown. (B–J) Body weight (B), oxygen consumption (C), locomotor activity (D), energy expenditure (E), body composition (F), adipocyte area (G), average food intake (H), respiratory exchange rate (RER) (I), and glucose tolerance test and area under the curve (AUC) (J) of adult *Nrp2*^loxP/loxP^ (control) and *Pomc*-Cre; *Nrp2*^loxP/loxP^ (mutant) mice. (K) Microphotographs and quantification of the density of α-melanocyte-stimulating hormone (αMSH)-immunoreactive (IR) fibers innervating the neuroendocrine paraventricular nucleus of the hypothalamus (PVHpml and PVHmpd), pre-autonomic PVH (postPVH), and DMH of adult *Nrp2*^loxP/loxP^ and *Pomc*-Cre; *Nrp2*^loxP/loxP^ mice. (L) Relative expression of corticotropin-releasing factor (*Crh*), thyrotropin-releasing hormone (*Trh*), and oxytocin (*Oxt*) mRNA in the PVN of the hypothalamus of adult *Nrp2*^loxP/loxP^ and *Pomc*-Cre; *Nrp2*^loxP/loxP^ mice. (M) Microphotographs and quantification of Pomc-expressing neurons and relative levels of Pomc mRNA in the ARH of adult *Nrp2*^loxP/loxP^ and *Pomc*-Cre; *Nrp2*^loxP/loxP^ mice. Data represented as mean ± SEM. ^∗^p < 0.05; ^∗∗^p < 0.01 versus *Nrp2*^loxP/loxP^. ARH, arcuate nucleus of the hypothalamus; DMH, dorsomedial nucleus of the hypothalamus; PVH, paraventricular nucleus of the hypothalamus; PVHmpd, dorsal component of the medial parvicellular PVH; PVHpml, lateral magnocellular PVH; post PVH, posterior part of the PVH; V3, third ventricle. Scale bars, 100 μm. See also Figure S5.

In the ARH, *Nrp2* mRNA expression was 2 times higher than that of *Nrp1* (Figure 4A). To investigate the role of Nrp2 in Pomc neurons *in vivo*, we crossed mice carrying a *Nrp2*^loxP^ allele (Walz et al., 2002) with mice expressing Cre recombinase in a *Pomc*-specific manner (*Pomc*-Cre) (Balthasar et al., 2004) to generate mice that lack *Nrp2* in Pomc-derived neurons. We repeated the *in vitro* co-culture assay with an ARH explant derived from *Nrp2*^loxP/loxP^ mice that received intra-ARH injections of an AAV-Cre vector and a PVH explant derived from WT mice. Genetic loss of Nrp2 in ARH neurons also blocked growth of axons toward the PVH (Figure 4F). As expected, the levels of *Nrp2* mRNA were decreased in the arcuate nucleus of *Pomc*-Cre; *Nrp2*^loxP/loxP^ mice whereas the levels of *Nrp1* were comparable between control and mutant mice (Figure S5A); there was a 3-fold reduction in *Nrp2* mRNA in sorted POMC neurons derived from mutant mice (Figure 5A). In contrast, there was no significant change in the levels of *Nrp1* and *Nrp2* mRNAs in the pituitary of mutant mice (Figure S5B and S5C), or in other hypothalamic nuclei and extra-hypothalamic brain regions (Figure S5D).

*Pomc*-Cre; *Nrp2*^loxP/loxP^ mice were born normally and had body weights indistinguishable from control littermates until 11 weeks of age (Figure 5B) when mutant mice displayed significantly higher body weights (Figure 5B). Oxygen consumption (VO_2_), locomotor activity and energy expenditure were reduced in mutant mice compared with control *Nrp2*^*l*oxP/loxP^ mice (Figures 5C–5E). Although there was no change in body composition (Figure 5F), there was an increase in adipocyte size in mutant mice (Figure 5G). Food intake and respiratory exchange ratio were not significantly different compared to controls (Figures 5H and 5I). *Pomc*-Cre; *Nrp2*^loxP/loxP^ mice displayed elevated levels of glucose 15 min after a glucose challenge, compared to control mice (Figure 5J). Leptin levels, but not insulin, T3, T4, and corticosterone levels, were significantly elevated in mutant mice (Figures S5E–S5I).

Although the overall distribution of αMSH-IR fibers was similar between mutant and control mice, the density of αMSH-IR fibers innervating the neuroendocrine and pre-autonomic parts of the PVH of *Pomc*-Cre; *Nrp2*^loxP/loxP^ mice was 2- to 3-fold lower than that observed in the *Nrp2*^loxP/loxP^ mice (Figure 5K). The density of αMSH projections to the DMH (Figure 5K) as well as to other major terminal fields (Figure S5J) was comparable between mutant and control mice. Corticotrophin-releasing hormone (*Crh)* and thyrotrophin-releasing hormone (*Trh*) expression in the PVH was increased, but *oxytocin* mRNA levels were unchanged (Figure 5L). The number of *Pomc* mRNA-expressing cells in the ARH of *Pomc*-Cre; *Nrp2*^loxP/loxP^ mice was comparable to that of control mice (Figures 5M and S5K–S5M). Together, these data indicate that Nrp2 signaling directs the formation of Pomc projections to the PVH with marked target specificity.

## Discussion

In this study, we identified rare heterozygous variants in *SEMA3s*, their receptors and co-receptors in individuals with severe early-onset obesity. In zebrafish, we showed that deletion of several genes in this pathway increased weight-related phenotypes establishing a role for these molecules in energy homeostasis. These genes might modulate body weight and/or fat mass by several potential mechanisms. In mice, we showed that Sema3s acting via Nrp2 direct the development of Pomc projections from the arcuate to the paraventricular nucleus of the hypothalamus. A role for Nrp1-mediated signaling in pre-adipocyte differentiation has been demonstrated (Ceccarelli et al., 2018). In zebrafish, *plxnd1* has been shown to regulate adipose tissue growth by affecting the formation of the extracellular matrix (Minchin et al., 2015), indicating that Semaphorin 3 signaling may affect non-neural mechanisms that contribute to adiposity.

### Rare Variants in the *SEMA3s*, Their Receptors, and Co-receptors

We found multiple rare variants in the genes encoding ligands, receptors, and co-receptors involved in Sema3 signaling by studying severely obese individuals. Variants were not fully penetrant and did not segregate with severe obesity in families demonstrating non-Mendelian inheritance. There are parallels with hypogonadotropic hypogonadism, where incomplete penetrance and variable expressivity within and across families has been observed (Cassatella et al., 2018). As the number of genes implicated in hypogonadotropic hypogonadism has increased, it has become clear that oligogenic inheritance (i.e., more than one gene mutated in the same individual) can in part explain these observations. Indeed, heterozygous variants in *SEMA3A*, *SEMA3E*, and *PLXNA1* can contribute to hypogonadotropic hypogonadism in an oligogenic manner with variable penetrance (Cariboni et al., 2015, Hanchate et al., 2012). In this study, while variant carriers did not carry additional variants in known obesity genes, it is plausible that other, as yet unidentified, genes may contribute to expression of the obesity phenotype in the individuals studied here.

Many challenges when studying rare variants might contribute to complex traits that do not follow Mendelian patterns of inheritance (Agarwala et al., 2013, Zuk et al., 2014). Statistical burden association tests are used to test for a difference in the load of rare variants predicted to have a functional impact in cases versus controls. Using this approach, we found nominal enrichment for very rare variants in the cluster of genes encoding SEMA3 ligands, receptors, and co-receptors when severely obese cases were compared to healthy controls indicating that rare variants within this gene-set may be associated with obesity. However, given the number of rare variants, their presence in cases and in controls, and the complexity of Sema3 signaling, larger genetic studies are needed to more fully test whether the burden of functionally significant variants (as tested in cells) is increased in severely obese individuals compared to controls and whether this association is driven by specific genes/combinations of genes.

Some of the neurodevelopmental phenotypes observed in variant carriers overlap with those seen in animals (Table 1). One male carrying *PLXNA3* D1710N had hypogonadotrophic hypogonadism. As *PLXNA3* lies on the X chromosome, this individual would have minimal residual signaling through *PLXNA3*. The true prevalence of hypogonadotrophic hypogonadism among variant carriers may be underestimated here as several probands were pre-pubertal children.

SEMA3 signaling is known to regulate the development of the enteric nervous system in rodents and rare heterozygous loss of function variants in *SEMA3C* and *SEMA3D* have been associated with Hirschsprung’s disease in humans (Jiang et al., 2015), a disorder characterized by failure of development of parasympathetic ganglion cells in the large intestine. Five people had severe medication-resistant constipation in childhood with multiple hospital admissions; two individuals had been investigated for Hirschsprung’s disease and one required a colostomy for severe dysmotility (*SEMA3D* D640Y). Additional studies in neuronal cells, experimental animal models of human variants, and in variant carriers will be needed to directly test the functional impact of these variants on the development of the enteric nervous system.

### Insights into the Molecular Mechanisms Disrupted by Rare Human Variants

Many of the SEMA3 variants reduced secretion and/or receptor-mediated signaling. In blinded studies, four mutants that had functional consequences on secretion and/or signaling in cells affected the density of neuronal projections, while the one WT-like variant (SEMA3D D640Y) studied, did not affect neuronal projections (Table S3). Paradoxically, some mutants exhibited increased secretion in cells. Experiments in chick DRGs have shown that the concentration of SEMA3s can determine which signaling pathway is activated and thereby influence the extent of growth cone collapse within a < 100 ng/mL to > 625 ng/mL range (Manns et al., 2012). As such, mutants that both increase and decrease secretion might disrupt axon guidance *in vivo*. Understanding how ligand concentration (as altered by human variants) affects axon growth may provide insights into the development of hypothalamic and extra-hypothalamic circuits in the human brain.

Mutant PLXN receptors were predominantly found within the endoplasmic reticulum rather than at the cell surface; structural modeling of the 21 mutants in *PLXNAs* and *NRPs* suggested several explanations for the misfolding of mutant receptors. The downstream consequences of aberrant receptor expression and signaling are challenging to predict as different NRP-PLXN complexes mediate signaling by different combinations of Semaphorins in different brain regions. For example, experiments in neurons suggest that Sema3A signals via Nrp1-PlxnA4 complexes, whereas Nrp2-PlxnA3 complexes mediate responses to Sema3F. Studies in *PlxnA3*-null mice show that PlxnA3 can mediate the effects of Sema3A and Sema3F (Cheng et al., 2001). However, neurons from PlxnA3-null mice only partially lose responses to Sema3A implying a degree of compensation through other receptor complexes, whereas hippocampal neurons from these mice lose essentially all responsiveness to Sema3F implying there is no redundant receptor for this specific response.

In preliminary studies, we modeled the effects of two NRP1 and –2 variants on melanocortin neural circuits in zebrafish. Compared to WT NRP2, A506V NRP2 resulted in a significant further reduction in the density of α-MSH-labeled fibers in the preoptic area (teleost homolog of the PVH) (Figure S3C). Compared to WT NRP1, the R237Q NRP1 mutant resulted in a further reduction in the density of AgRP immunoreactive fibers innnervating the anterior tuberal nucleus (teleost homolog of the VMH) (Figure S3C). Given the challenges associated with dissecting the impact of these molecules on neuronal circuits and weight-related phenotypes, deletion/reactivation studies of components of the signaling pathway in specific neuronal populations will be needed to determine the extent to which other components of the signaling complex can compensate for the partial lack of expression of mutant receptors *in vivo*.

### Role of SEMA3 Signaling in the Development of Hypothalamic Neural Circuits

Our data suggest that Sema3 signaling in developing Pomc neurons contributes to the regulation of energy homeostasis and glucose homeostasis. The relatively modest effect of *Nrp2* deletion in Pomc neurons on body weight is not surprising, as only Pomc axonal projections to the PVH were disrupted in this mouse model. Our findings align with experiments specifically disrupting leptin signaling in ARH Pomc neurons (Berglund et al., 2012). Further in-depth exploration of the degree of compensation/plasticity associated with targeted experimental manipulations will be needed. Further characterization of the impact of human variants in mature neurons, where they can affect the maturation and density of dendritic spines, synaptogenesis, and synaptic plasticity (Orr et al., 2017), may inform understanding of the mechanisms that underlie human disorders characterized by hypothalamic dysfunction.

## STAR★Methods

### Key Resources Table

**Table:** 

REAGENT or RESOURCE	SOURCE	IDENTIFIER
**Antibodies**		

Flag	Sigma-Aldrich	Cat# F3040; RRID: AB_439712
Glu-Glu	Covance	Cat# PEP-115P; RRID: AB_291283
Anti-Mouse IgG-HRP	Bio Rad	Cat #1721011; RRID: AB_11125936
Anti-rabbit IgG-HRP	Dako	Cat# P0448; RRID: AB_2617138
Biotin anti-human IgG	Sigma-Aldrich	Cat# SAB3701279 ; RRID: AB_AB_258559
Beta-actin	Cell Signaling Technology	Cat #4970; RRID: AB_2223172
Calnexin	Cell Signaling Technology	Cat #2679; RRID: AB_2228381
Anti-mouse AF488	Invitrogen	Cat#A28175; RRID: AB_2536161
Anti-rabbit AF568	Invitrogen	Cat#A10042; RRID: AB_2534017
Human Neuropilin 1	R&D Systems	Cat#AF3870; RRID: AB_884367
Human Neuropilin 2	R&D Systems	Cat#MAB2215; RRID: AB_2155370
betaIII tubulin	Covance	Cat#MRB-435P; RRID: AB_663339
Neuropeptide Y	Chemicon	Cat#AB1583; RRID: AB_2236176
α-MSH	Millipore	Cat#AB5087; RRID: AB_91683
Digoxigenin	Roche	Cat# 11093274910; RRID: AB_514497
Perilipin A	Sigma-Aldrich	Cat#P1873-200UL; RRID: AB_532267

**Bacterial and Virus Strains**		

Subcloning Efficiency DH5α Competent Cells	Thermo Fisher Scientific	Cat#18265017
XL10-Gold	Agilent	Cat# 200315
AAV5-CMV.HI.eGFP-Cre.WPRE.SV40	This paper	NA

**Biological Samples**		

Hypothalamus (human fetus at 14 weeks)	Dr Prevot, Inserm U1172, Lille, France	NA
Hypothalamus (human, 19 years ± 1.5 years) n = 2	Dr Prevot, Inserm U1172, Lille, France	NA

**Chemicals, Peptides, and Recombinant Proteins**		

Lipofectamine 2000	Invitrogen	Cat#11668027
Semaphorin 3C	R&D Systems	Cat#5570-S3-050
Wheat Germ Agglutinin, Alexa Fluor 647 Conjugate	Invitrogen	Cat# W32466

**Critical Commercial Assays**		

T7 Endonuclease I (T7E1)	New England Biolabs	Cat#M0302
QuantaBlu Fluorogenic Peroxidase Substrate Kit	Thermo Fisher Scientific	Cat#15169
PicoPure RNA Isolation Kit	Thermo Fisher Scientific	Cat# KIT0214
RNeasy Lipid Tissue Mini Kit	QIAGEN	Cat#74804
High-Capacity cDNA Reverse Transcription Kit	Thermo Fisher Scientific	Cat#4368814

**Experimental Models: Cell Lines**		

HEK293	ATCC	CRL-1573
COS-7	ATCC	CRL-1651
U87MG	Gift from Gera Neufeld	NA
HUVEC	Gift from Gera Neufeld	NA

**Experimental Models: Organisms/Strains**		

Zebrafish: WIK		ZDB-GENO-010531-2
Mouse (C57BL/6): *Pomc*-Cre	Balthasar et al., 2004	NA
Mouse (C57BL/6): ROSA-TdTomato reporter line	Madisen et al., 2010	NA
Mouse (C57BL/6): Pomc-Cre; Nrp2 loxP/loxP	This Paper	NA
Mouse (C57BL/6): Nrp2 loxP/loxP	Walz et al., 2002	NA

**Recombinant DNA**		

Human Sema3A expression plasmid	OriGene	CAT#: RC213681
Human Sema3B expression plasmid	OriGene	CAT#: RC223532
Human Sema3C expression plasmid	OriGene	CAT#: RC205269
Human Sema3D expression plasmid	OriGene	CAT#: RC216032
Human Sema3E expression plasmid	OriGene	CAT#: RC216038
Human Sema3F expression plasmid	OriGene	CAT#: RC208333
Human Sema3G expression plasmid	OriGene	CAT#: RC222035
Human PlexinA1 expression plasmid	OriGene	CAT#: RC222057
Human PlexinA2 expression plasmid	OriGene	CAT#: RC221024
Human PlexinA3 expression plasmid	OriGene	CAT#: RC212456
Human PlexinA4 expression plasmid	OriGene	CAT#: RC226436
Human Neuropilin 1 expression plasmid	OriGene	CAT#: RC217035
Human Neuropilin 2 expression plasmid	OriGene	CAT#: RC220920

**Software and Algorithms**		

Ensembl GeneTree	https://uswest.ensembl.org/Help/View?id=137	ENSGT00760000119048
Clustal Omega	https://www.ebi.ac.uk/Tools/msa/clustalo/	NA
Unrooted	http://pbil.univ-lyon1.fr/software/unrooted.html	NA
DanRer 10	Varshney et al., 2015	NA
TIDE	https://tide.nki.nl/	NA
VerifyBamID (v1.0)	http://csg.sph.umich.edu/kang/verifyBamID/	NA
EIGENSTRAT v4.2	Price et al., 2006	NA
PLINK v1.07	https://www.cog-genomics.org/plink2	NA
Fiji	https://fiji.sc/	NA
Prism	https://www.graphpad.com/scientific-software/prism/	NA
Modeler	https://salilab.org/modeller/	NA
Pymol	https://pymol.org/2/	NA

### Contact for Reagent and Resource Sharing

Further information and requests for resources and reagents should be directed to and will be fulfilled by the Lead Contact, I. Sadaf Farooqi (isf20@cam.ac.uk).

### Experimental Model and Subject Details

#### Studies in humans

The Genetics of Obesity Study (GOOS) is a cohort of 7,000 individuals with severe early-onset obesity; age of obesity onset is less than 10 years (Farooqi et al., 2003, Wheeler et al., 2013). Severe obesity is defined as a body mass index (weight in kilograms divided by the square of the height in meters) standard deviation score greater than 3 (standard deviation scores calculated according to the United Kingdom reference population). The mean age of the subjects with variants was 13.0 ± 1.6 years. 28 were female and 11 were male, mean BMI or BMI SDS did not differ between males and females (35.3 ± 3.4 kg/m^2^ versus 34.9 ± 1.8 kg/m^2^; 3.8 ± 0.2 SDS versus 3.8 ± 0.2 SDS). All studies were approved by the Cambridge Local Research Ethics Committee and each subject (or their parent for those under 16 years) provided written informed consent; minors provided oral consent. Healthy blood donors from the INTERVAL project were used as controls (Moore et al., 2014). All participants gave informed written consent.

#### Studies in cellular models

HEK293 (XX female) and COS7 (XY male) cells were cultured in high glucose Dulbecco’s modified eagle medium (DMEM, GIBCO, 41965) supplemented with 10% fetal bovine serum (FBS, GIBCO, 10270, South America origin), 1% GlutaMAX (100X) (GIBCO, 35050), and 100 units/mL penicillin and 100 μg/mL streptomycin (Sigma-Aldrich, P0781). U87MG (XY male) cells were cultured in MEM with non-essential amino acids (Sigma) supplemented with 10% FBS (GIBCO), 1mM Sodium pyruvate, 1% GlutaMAX (100X) (GIBCO, 35050), and 100 units/mL penicillin and 100 μg/mL streptomycin (Sigma-Aldrich, P0781). HUVECs (XX, female) cells were cultured in M199 (with Glutamine) (Sigma-Aldrich, M4530), supplemented with 20% FBS (GIBCO), 1% GlutaMAX (100X) (GIBCO, 35050), 100 units/mL penicillin and 100 μg/mL streptomycin (Sigma-Aldrich, P0781), 1% Vitamins- MEM-EAGLE Vitamin Solution (GIBCO, 11120052). HUVEC cells were grown in flasks coated with gelatin (Sigma-Aldrich, G1890). U87MG and HUVEC cells were a kind gift from Gera Neufeld (the Rappaport Institute). Cells were incubated at 37°C in humidified air containing 5% CO_2_ and transfections were performed using Lipofectamine 2000 (GIBCO, 11668) in serum-free Opti-MEM I medium (GIBCO, 31985) according to the manufacturer’s protocols.

#### Studies in zebrafish

All zebrafish experiments were conducted in accordance with the Animals (Scientific Procedures) Act 1986, and following UK Home Office approval (License #: P0662C816). Using CRISPR/Cas9, deletion mutants of zebrafish homologs of Semaphorin3s, PlexinA1-4 and Nrp1-2 were generated and characterized by their length and weight. Zebrafish were reared at equivalent densities and the analyses were conducted prior to any overt sexual differentiation.

#### Studies in mice

All animal procedures were conducted in compliance with and approved by the IACUC of the Saban Research Institute of the Children’s Hospital of Los Angeles. Animals were housed under specific pathogen-free conditions, maintained in a temperature-controlled room with a 12 h light/dark cycle, and provided *ad libitum* access to water and standard laboratory chow (Special Diet Services). To genetically label Pomc fibers for *in vitro* studies, *Pomc*-Cre mice (Balthasar et al., 2004) were crossed with a ROSA-TdTomato reporter line (Madisen et al., 2010). To generate Pomc-specific Nrp2 knockout (*Pomc*-Cre; *Nrp2*^loxP/loxP^) mice, *Pomc*-Cre mice were mated to mice carrying a loxP-flanked Nrp2 allele (*Nrp2*^loxP/loxP^) (Walz et al., 2002). Breeding colonies were maintained by mating *Pomc*-Cre; *Nrp2*^loxP/+^ mice to *Nrp2*^loxP/loxP^ mice. Cre-negative *Nrp2*^loxP/loxP^ were used as controls. All mice were generated in a C57BL/6 background and only male mice were studied.

### Method Details

#### Studies in Humans

##### Sequencing, variant calling, and quality control

Details about sequencing and variant calling for the severe childhood onset obesity project (SCOOP, UK individuals of European ancestry recruited to the GOOS cohort) cases (Hendricks et al., 2017), as part of the UK10K exomes, and the INTERVAL controls have been reported previously (Singh et al., 2016). Briefly, single–sample variant calling using GATK Haplotype Caller (v3.2) was performed on the union of Agilent v3 and v5 targets plus a 100 base pair flanking region on 9795 UK10K and INTERVAL samples, including SCOOP cases (N = 982) and INTERVAL controls (N = 4499). The called variants were then merged into 200 sample batches and were joint-called using Genotype VCFs and default settings (DePristo et al., 2011, Van der Auwera et al., 2013). To ensure high-quality variant calls across all datasets and sequencing batches, only variants with at least 7x coverage in at least 80% of samples were called. We applied further variants QC keeping only variants with a calibrated VQSR tranche above 99.75% sensitivity, missingness < 20%, Hardy-Weinberg equilibrium χ2 p value > 10E-8, mean genotype quality ≥ 30, and variants in low-complexity regions as described previously (Li, 2014). Further, individual genotypes were set to missing if any of the following was true: GQ < 30, alternate allele read depth (DP1) < 2, allelic balance (AB) < 0.2, or AB > 0.8.We used VerifyBamID (v1.0) (Jun et al., 2012) and a threshold of ≥ 3% to identify contaminated samples, principal components calculated from the 1000Genomes Phase I integrated call set (Abecasis et al., 2010) using EIGENSTRAT v4.2 (Price et al., 2006) to identify non-Europeans, and pairwise identity by descent estimates from PLINK v1.07 (Purcell et al., 2007) with a threshold of ≥ 0.125 to identify related individuals. Contaminated, non-European, and related samples were removed resulting in 927 SCOOP cases and 4,057 INTERVAL controls for analysis.

##### Gene-set enrichment

We performed gene-set enrichment similar to previous analyses described in (Purcell et al., 2014, Singh et al., 2016). Briefly, using PLINK/SEQ (Purcell et al., 2007) we calculated individual gene region burden test-statistics for an enrichment in cases compared to controls of very rare (MAF < 0.025%) variants meeting one of two predicted functional requirements: functional or loss of function, or only loss of function. We then used the SMP utility to calculate the gene set enrichment while controlling for exome-wide differences between cases and controls. Twenty thousand case control permutations were used to estimate the empirical gene set enrichment p value on which a Bonferroni adjustment for two tests (i.e., functional and LoF, and LoF only) was applied to arrive at the adjusted p value.

#### Functional Characterization of Rare Human Variants

##### Cloning and site-directed mutagenesis

The cDNA constructs used throughout the study were made by site-directed mutagenesis using QuikChange II XL kit (Agilent Technologies, 200516) according to the manufacturer’s protocols. SEMA3s cDNA constructs contained a C-terminal Myc/DDK tag; NRP1-2 and PLXNA1-4 cDNA constructs contained a N-terminal Glu-Glu tag and C-terminal Myc/DDK tag; all constructs were ligated into pCMV6-Entry vector (Origene) using Sgf I/Mlu I site. All constructs were verified by Sanger sequencing.

##### SEMA3s secretion, cell surface and total cell ELISA

Secreted SEMA3s were detected in the medium of transfected HEK293 cells grown in 96 well plates. Medium from these cells was transferred to MaxiSorp plates and bound SEMA3 immunodetected with an anti-Flag antibody. Cell surface antigen was quantified on transfected non-permeabilised live HEK293 cells in 96 well plates using an anti-Glu-Glu antibody. Total cell antigen was detected in transfected HEK293 cells in 96-well plates after fixation and permeabilisation using an anti-Flag antibody.

##### Secreted Semaphorin ELISA

50,000 HEK293 cells were seeded into each well of a poly-D-lysine coated 96-well plate. After 24hr the cell medium was exchanged for Opti-MEM (GIBCO) and cells transfected. Cell medium was exchanged once more with 50 μL of Opti-MEM containing a 1:400 dilution of Protease inhibitors (Sigma). 48 h after transfection, cell medium from two identically transfected 96-well plates was pooled together (100 μl total volume) with the addition of 25mM HEPES pH7.5 and centrifuged at 1500rpm for 5 min. The top 80 μL of medium was transferred to black MaxiSorp plates (NUNC), the plates were sealed and incubated overnight at 4^ο^C with gentle agitation. Cell medium was removed and plates were washed thrice for 10 min with gentle agitation before blocking and immunodetection as described in the ELISA procedure below using the anti-Flag antibody and QuantaBlu Fluorogenic Peroxidase Substrate Kit (Thermo).

##### Cell surface and total protein ELISA

40,000 HEK293 cells were seeded into each well of a poly-D-lysine coated 96-well plate and transfected the following day. 48 h after transfection cells were washed once with PBS and processed for the detection of either cell surface or total cellular antigens. Cell surface protein was detected by blocking live cells on ice (3% dry milk, 50mM Tris, pH 7.4 in PBS) for 30 mins and then incubation with anti-Glu-Glu antibody (Covance) in blocking buffer on ice for 2 hr. Cells were washed thrice for 10 min on ice with PBS and then fixed (3.7% formaldehyde in PBS) for 10 min on ice and then 10 min at room temperature. Cells were washed thrice for 10 min with PBS, blocked again for 30 min and incubated with secondary antibody as described for the total cellular antigen detection procedure. Total cellular antigens were detected in fixed (3.7% formaldehyde in PBS) and permeabilised (0.1% triton in PBS for 5 mins) cells after blocking for 1hr and incubation with anti-flag antibody (Sigma) for 2 hr in blocking buffer. Cells were washed thrice for 10 min with PBS, incubated with anti-mouse HRP antibody (BioRad) (1.5% dry milk, 50mM Tris, pH 7.4 in PBS) for 1 hr, washed thrice more for 10 min with PBS before incubation with HRP substrate (TMB, BioRad) and measurement of absorbance at 450nM.

##### Cell collapse assays

SEMA3-induced cell collapse was tested in U87MG cells grown on a gelatin coated surface. Cells were exposed to SEMA3-conditioned cell medium and collapsed cells micrographed after 30 min and counted manually. Receptor mutants were tested by transfecting COS7 cells with *PLXNA*, *NRP*, and GFP, followed by exposure to purified SEMA3C at 2 μg /ml for 30 min. Collapsed cells were micrographed under blue laser and collapse quantified using ImageJ.

Semaphorin expression vectors were transfected into HEK293 cells at 60%–90% confluency in 10cm dishes. Growth medium was replaced with serum-free medium after 12 h. After 48 h the medium, containing secreted semaphorin, was harvested, aliquoted, and snap frozen in liquid nitrogen followed by storage at −80°C. The cell contraction assay with mutant ligands was carried out as reported previously (Sabag et al., 2014). U87MG cells were used for SEMA3A, SEMA3B, SEMA3D, SEMA3F, and SEMA3G induced collapse. HUVEC cells were used with SEMA3C and SEMA3E. The morning of the assay 10^5^ cells/well were seeded into a 12-well plate coated with gelatin (Sigma, UK). Cells were allowed to attach to the plate surface for 5 h. Semaphorin-containing medium was then added to the wells at a 1:2 dilution and cells kept at 37°C for 30 min. Following collapse cells the plates were photographed under the microscope and collapsed cells counted individually (at least 100 cells per well). To test cell collapse mediated by mutant receptors, COS7 cells were seeded at 2x10^5^ cells/well in a gelatin coated 6-well plate, and the following day co-transfected with PlxnA1-4, Nrp1-2, and GFP. After 24 h purified, recombinant, human SEMA3C (R&D Systems, UK) was added at a concentration of 2 μg/ml and incubated at 37°C for 30 min. Following collapse, cells were photographed under the microscope and collapsed cells were counted using image recognition software on ImageJ (NIH, Bethesda). Collapse efficiency was assessed by counting the proportion of collapsed cells 30 min following addition of the WT semaphorin to the culture medium.

##### Ligand binding assay

Cells expressing *NRP1-2* or co-expressing *NRP1-2* and *PLXNA1-4* were incubated with recombinant human SEMA3C-Fc chimera and binding was quantified using an anti-human IgG (Fc specific) antibody. HEK293 cells (40,000 cells/well) were seeded in a poly-D-lysine coated 96-well plate. After 24 h, 70%–80% confluent cells were transiently transfected with WT or mutant NRP1/2 alone or co-transfected with NRP1/2 (WT and mutant) and WT PLXNA1-4. For NRP and PLXNA constructs, the amount of DNA used was 30 and 60 ng, respectively. Twenty four hours after transfection, cells were blocked with 1% BSA in serum-free media (SFM) for 30 min at 37°C then incubated with recombinant human SEMA3C Fc chimera (0.2 – 10 nM) (rhSEMA3c-Fc, R&D System, catalog number 5570-S3-050) in SFM supplemented with 1% BSA for 30 min at 37°C. Following 5 washes with Dulbecco’s Phosphate Buffered Saline (DPBS), cells were fixed with 3.7% formaldehyde at room temperature (RT) for 15 min, washed three times with PBS and subsequently blocked for 30 min at room temperature (1% BSA in 50 mM Tris-Phosphate buffer pH 7.4). For binding detection, a biotinylated goat-anti-human IgG (Fc specific) (Sigma-Aldrich, SAB3701279) diluted 1:10000 in blocking buffer was incubated for 1 h at RT. Subsequently, the wells were washed three times with PBS and a peroxidase-conjugated Streptavidin (Thermo) (dilution 1:12000) was added to the wells and incubated for 30 min at RT. After three washes, bound SEMA3C was quantified by the addition of 90 μL of TMB (Biorad) per well, and stopped by 60 μL of 0.2M H_2_SO_4_. Absorbance at 450 nm was measured using an Infinite M1000 PRO microplate reader (Tecan). Specific binding was determined by subtracting non-specific binding (cells transfected with empty vector [pCMV6]) from total binding. Specific binding was plotted and *k*_*d*_ and *B*_*max*_ determined using Prism 6.07 (GraphPad) software.

##### Reducing and non-reducing Immunoblotting

HEK293 cells were seeded in 10 cm dishes and transfected the following day once the cells had reached 90% confluency. 6 h after transfection the cell medium was replaced with serum-free medium containing 1:400 dilution of protease inhibitors. 48 hr after transfection the cell medium was supplemented with additional protease inhibitors. 72 hr after transfection the cell medium was harvested from the dishes, centrifuged at 1500 rpm for 5 min and the sample was prepared for electrophoresis (resuspended in 1x BOLT LDS sample buffer (Thermo) and 1x Bolt reducing agent (Thermo) and heated for 10mins at 70^ο^C). Cells were washed once with PBS and lysed in triton lysis buffer (50 mM Tris pH7.5, 150 mM NaCl, 1 mM EGTA, 1 mM EDTA, 1 mM sodium orthovanadate, 50 mM sodium fluoride, 10 mM sodium pyrophosphate, 10 mM sodium glycerophosphate, 1% (v/v) Triton X-100 and protease inhibitors (Roche). Lysates were centrifuged at 14,000 rpm for 20 min and the protein concentration determined using a Bradford assay (BioRad). Equal amounts of protein were prepared for electrophoresis, as described above. For non-reducing Immunoblotting, samples were prepared in 1x BOLT LDS sample buffer (without reducing agent) and heated at 60^ο^C for 5 min. Protein electrophoresis was performed using BOLT gels (Thermo) and transfer onto nitrocellulose using an iBLOT (Thermo). Membranes were probed overnight at 4^ο^C using an anti-Flag primary (Sigma) and anti-mouse HRP (DAKO) secondary antibody or an anti-actin (NEB) primary and anti-rabbit secondary antibody (DAKO).

##### Immunofluorescence and Confocal Microscopy

80,000 COS7 cells were seeded onto glass coverslips in 12-well plates and transfected. 48 h after transfection cells were fixed with 4% formaldehyde in PBS for 10 min and washed three times for 5 min with PBS before membrane staining with 10 μg/ml Alexa Fluor 647 conjugated wheat germ agglutinin for 10 min. Cells were washed three more times, permeabilised with 0.1% Triton X-100 in PBS for 5 min before being washing again. Cells were incubated in blocking buffer (3% BSA in PBS) for 1h, then primary antibody (mouse anti-Flag and Rabbit anti-Calnexin (Cell signaling) diluted in blocking buffer followed by washing and incubation with secondary antibody (anti-mouse Alexa Fluor 488 and anti-Rabbit Alexa Fluor 568 (ThermoFisher). Cells were washed and incubated with DAPI (1 μg/ml) for 5 min before being washed again and mounting onto coverslips using mounting medium (VECTASHIELD with DAPI). Slides were imaged using a Leica SP8 confocal microscope with a 63x objective (NA 1.4) and images processed using FIJI.

##### Structural analysis

To study how the human *SEMA3-PLXNA-NRP* variants affect signaling, we mapped the mutated residues onto structural models and assessed their potential effects on stability and protein-protein interactions. For SEMA3s, all human SEMA3 sequences (SEMA3A-3G) were first aligned to the sequence of mouse SEMA3A. A homology model encompassing the first three Sema3 domains, the sema, PSI and Ig domains, was made by combining the sema-PSI segments of the mouse Sema3A crystal structure at 3.3 Å resolution (PDB: 4GZ8) together with a homology model of the Sema3 Ig domain generated using the 3.0 Å crystal structure of human Sema4D which includes a more complete Sema-PSI-Ig segment (PDB: 1OL2). The human *SEMA3* variants were then mapped onto the equivalent residues in the mouse Sema3A model. To locate the variants potentially affecting the Sema3-Plxn-Nrp interfaces, the crystal structure of mouse Sema3A-PlxnA2-Nrp1 (PDB: 4GZA) was used as a reference. Similarly, human PlxnA1-A4 sequences were aligned with mouse PlxnA1 and the mouse residues corresponding to the human variants were identified. The human *PLXN* variants on the extracellular segments (domains sema to IPT6) were mapped onto the 4 Å crystal structure of the mouse PlxnA1 ectodomain (PDB: 5L56). Variants in the PlxnA cytoplasmic domain were mapped onto a homology model of PlxnA1 generated using the 3.3 Å crystal structure of the zebra fish PlxnC1 active dimer (PDB: 4M8M). Human *NRP1* and *NRP2* variants were mapped onto the equivalent positions in the 2.7 Å crystal structure of mouse Nrp1 (PDB: 4GZ9). The MAM domain of the Nrps was not modeled. All protein sequences were obtained from the UniProt database (http://www.uniprot.org/). Protein sequence alignment was performed using the ClustalW Omega server (https://www.ebi.ac.uk/Tools/msa/clustalo/). Homology models were generated and analyzed using Modeler. Structural visualization and image production was performed using Pymol (https://pymol.org/2/).

#### Studies in Zebrafish

Zebrafish homologs of class 3 Semaphorins, class A Plexins and Nrps were identified using Ensembl GeneTree (ENSGT00760000119048). Phylogenetic trees were constructed from the zebrafish, human, and mouse orthologs using Clustal Omega (Sievers et al., 2011). Phylogenetic trees were visualized using the Unrooted software (http://pbil.univ-lyon1.fr/software/unrooted.html). Two-five non-overlapping short guide RNAs (gRNA) targeting each zebrafish gene were identified using the Zebrafish Genomics track data hub (GA targets) in DanRer 10; gRNA templates were assembled and mRNA produced as described (Varshney et al., 2015) with the exception that the T7 promoter was substituted with an SP6 promoter. sgRNAs (200 ng each) were incubated with 600 ng Cas9 protein (New England Biolabs, #M0646T) for 5 min at 37°C prior to 2 nL being injected into 1-cell stage zebrafish WIK embryos. For each gRNA, mutagenesis was confirmed using T7 Endonuclease I (T7E1, New England Biolabs, #M0302), and quantified using TIDE (Brinkman et al., 2014). gRNA and primer sequences for T7 assays are available from JENM on request. Zebrafish were reared at equivalent densities. Zebrafish injected with Cas9 protein only were used as the control group. However, gRNA-only injected zebrafish were also measured and no difference in somatic growth was observed between Cas9-only fish. Length and weight were measured as previously described (Parichy et al., 2009). Nile Red staining on postembryonic larvae was conducted as described (Minchin et al., 2015). Percent body fat was calculated as previously described (Minchin and Rawls, 2017), in stage matched larvae. For the initial CRISPR screen, analyses were conducted at 5, 14 and 35 days post fertilization in three independent experiments. Between 6 and 25 animals were used for each group within an experiment. Analyses were conducted prior to any overt sexual differentiation. Standard length, weight and % body fat were analyzed in three additional *nrp2* experiments and used between 40 and 83 animals in total.

##### Immunohistochemical analysis of αMSH and AgRP projections

Human wild-type *NRP1* and *NRP2* along with the *NRP1* R237Q and *NRP2* A506V variants were cloned into the pCMV6-Entry vector (Origene), which was then linearized using the AgeI-HF restriction enzyme (New England Biolabs, #R3552), column cleaned (Zymo Research Clean & Concentrator, #D4013) and RNA transcribed using the mMessage mMachine T7 Transcription Kit as according to the manufacturers protocol (Thermo Fisher, #AM1344). Capped RNA was then cleaned using Zymo RNA Clean & Concentrator columns (Zymo Research Clean & Concentrator, #R1013) and 100 pg injected into one-cell stage wild-type (WIK) zebrafish embryos. Injected fish were raised at 20 fish/3L until 35 dpf. Thirty-five day old fish (n = 8-9/group) were fixed overnight with 4% paraformaldehyde/borate buffer. The fish brains were then frozen, sectioned at 20-um thick, and processed for immunofluorescence using standard procedures (Bouret et al., 2004). Briefly, sections were incubated with a sheep anti-αMSH antibody (1:40,000, Millipore) and a rabbit anti-AgRP (1:1,000, Phoenix Pharmaceuticals). The primary antibodies were visualized with Alexa Fluor 568 donkey anti-sheep IgG and Alexa Fluor 488 donkey anti-rabbit IgG (1:200, Millipore). Sections were counterstained using bis-benzamide (1:10,000, Invitrogen) to visualize cell nuclei. Two sections through the preoptic area (POA), anterior tuberal nucleus of hypothalamus (ATN), and lateral hypothalamic nucleus (LH) were acquired using a Zeiss LSM 710 confocal system equipped with a 20X objective. To quantify fibers density, each image plane was binarized to isolate labeled fibers from the background and to compensate for differences in fluorescence intensity. The integrated intensity, which reflects the total number of pixels in the binarized image, was then calculated for each image as previously described (Bouret et al., 2004). This procedure was conducted for each image stack.

#### Studies in Mice

##### Analysis of gene expression

The hypothalamus of E10, E12, and E14 mouse embryos (n = 3-4/group) as well as the ARH, DMH, LHA, POA, PVH, SCN, and VMH of P10 male mouse pups and the ARH and PVH of 8-weeks-old mice (n = 5/group) were dissected under a stereomicroscope. In addition, the hypothalamus of a human fetus at 14 weeks of gestational age and hypothalamic of human young adults (19.0 years ± 1.5 year; n = 2/group; generously provided by Dr Prevot, Inserm U1172, Lille France) was collected. Total RNA was isolated using the Arcturus PicoPure RNA isolation kit (for mouse tissues) (Life Technologies) or the RNeasy Lipid tissue kit (for human tissues) (QIAGEN). cDNA was generated with the high-capacity cDNA Reverse Transcription kit (Life Technologies). Quantitative real-time PCR was performed using TaqMan Fast Universal PCR Mastermix and the commercially available Taqman gene expression primers. mRNA expression was calculated using the 2^-ΔΔCt^ method after normalization to the expression of *Gapdh* housekeeping gene. All assays were performed using an Applied Biosystems 7900 HT real-time PCR system.

##### Real-time PCR primers

Real-time PCR was performed on Applied Biosystems 7900HT Fast Real-Time PCR System using TaqMan Gene Expression Assays (Applied Biosystems): *Pomc* (Mm00435874_m1), *Npy* (Mm03048253_m1), *Agrp* (Mm00475829_g1), *Leprb* (Mm00440181_m1), *Crh* (Mm01293920_s1), *Trh* (Mm01963590_s1), *Oxt* (Mm00726655_s1), *Nrp1* (Mm00435379_m1), *Nrp2* (Mm00803099_m1), *Plxna1* (Mm00501110_m1), *Plxna2* (Mm00801930_m1), *Plxna3* (Mm00501170_m1), *Plxna4* (Mm00558881_m1), *Sema3a* (Mm00436469_m1), *Sema3b* (Mm00436477_m1), *Sema3c* (Mm00443121_m1), *Sema3d* (Mm01224783_m1), *Sema3e* (Mm00441305_m1), *Sema3f* (Mm00441325_m1), *Sema3g* (Mm01219778_m1), *Gapdh* (Mm99999915_g1).

##### Signaling activity of wild-type and mutant SEMA3s in HEK293 cells

HEK293 cells were grown in monolayers in 5% CO2 at 37°C, in Dulbecco’s modified Eagle’s medium (Life Technologies) containing 1 mM sodium pyruvate, 2 mM glutamine, 50 mM glucose, and supplemented with 10% fetal bovine serum (Invitrogen), 100 μg/ml streptomycin and 100 U/ml penicillin. A cDNA containing the entire coding region of the human *SEMA3A, SEMA3B, SEMA3C, SEMA3D, SEMA3E, SEMA3F, SEMA3G* was inserted into a pRK5 plasmid expression vector. Recombinant plasmids containing *SEMA3* cDNAs harboring the variants identified in obese individuals were then engineered using the QuickChange mutagenesis protocol (Stratagene). HEK293 cells were transiently transfected using a fast-forward protocol (Lipofectamine 2000, Invitrogen). Conditioned medium was collected 48 h after transfection, tested for the presence of Flag by western blot analysis using an anti-Flag antibody (Sigma-Aldrich).

##### Explant co-culture assays

Brains were collected from P4 Pomc-Cre; TdTomato male mice (for ARH explants) and P8-P12 wild-type male mice (for PVH, DMH, and VMH explants) and sectioned at a 200-um thickness with a vibroslicer as previously described In addition, *Nrp2*^loxP/loxP^ mice received stereotaxic bilateral injections of AAV5-CMV.HI.eGFP-Cre.WPRE.SV40, in the ARH at P0. Control group consisted of WT mice that received bilateral stereotaxic injections of AAV5-CMV.HI.eGFP-Cre.WPRE.SV40 virus into the ARH at P0. Brains were collected for co-culture experiments at P6**.** The ARH, PVH, DMH, and VMH were then carefully dissected out of each section under a stereomicroscope. ARH explants were co-cultured onto a collagen matrix (BD Bioscience) with either PVH, DMH, or VMH explants (n = 4-12 explants/group from 3-6 independent experiments) or HEK293 cells transfected with Sema3-encoding vectors described above (n = 6-28 explants/group from 3-6 independent experiments). Control experiments included co-cultures with control HEK293 cell aggregates and cortical explants. For the heterochronic cultures, beginning on the first day *in vitro*, each explant were transferred to fresh modified Basal Medium Eagle (Invitrogen) containing either Nrp1 or Nrp2 neutralizing antibodies (1.5 ug/ml, R&D Systems) or control goat IgGs. After 48 h, the explants were fixed in paraformaldehyde and stained with betaIII tubulin (TUJ1 monoclonal antibody, 1:5,000, Covance) or NPY (1:3,000, Chemicon). Pomc-TdTomato^+^, TUJ1^+^ and NPY^+^ neurites extending from the ARH explants were analyzed as followed. Confocal image stacks of the co-cultures, that included the proximal and distal edges of the ARH explant, were acquired using a Zeiss LSM 710 confocal system equipped with a 10X objective. Slides were numerically coded to obscure the treatment group. Image analysis was performed using ImageJ analysis software (NIH). For the *in vitro* experiments, each image plane was binarized, and the total density of Pomc+, TUJ1+ and Npy+ neurites were measured in the proximal (P) and distal (D) parts of the explant. The P/D ratio is a measure of growth toward or away from the explant, with a ratio > 1 indicating net increase in projections and < 1 indicating decreased axon growth.

##### Immunohistochemical analysis of αMSH projections

15-17-weeks old male mice (n = 4/group) were perfused transcardially with 4% paraformaldehyde. The brains were then frozen, sectioned at 30-um thick, and processed for immunofluorescence using standard procedures (Bouret et al., 2004). Briefly, sections were incubated with a sheep anti-αMSH antibody (1:40,000, Millipore). The primary antibody was visualized with Alexa Fluor 488 donkey anti-sheep IgG (1:200, Millipore). Sections were counterstained using bis-benzamide (1:10,000, Invitrogen) to visualize cell nuclei. Two sections through various anatomical compartments of the PVH (PVHmpd and PVHpml, neuroendocrine; and postPVH, autonomic) were acquired using a Zeiss LSM 710 confocal system equipped with a 20X objective. For the quantitative analysis of fibers density, each image plane was binarized to isolate labeled fibers from the background and to compensate for differences in fluorescence intensity. The integrated intensity, which reflects the total number of pixels in the binarized image, was then calculated for each image. This procedure was conducted for each image stack.

##### Analysis of Pomc cell numbers

15-17-weeks old male mice (n = 4/group) were perfused transcardially with 4% paraformaldehyde. The brains were then frozen, sectioned at 30-um thick, and processed for fluorescent *in situ* hybridization using standard procedures. Two sections through the ARH were acquired using a Zeiss LSM 710 confocal system equipped with a 20X objective. For the quantitative analysis of cell number, the number of Pomc+ cell bodies in the ARH were manually counted using ImageJ analysis software (NIH). The average number of cells counted in two ARH hemi-sections from each mouse was used for statistical comparisons.

##### Physiological measurements in mice

Male mice (n ≥ 9/group) were weighed every 2 days from P4 to P22 (weaning) and weekly from 4 to 14 weeks using an analytical balance. Body composition analysis (fat/lean mass) was performed in 16-week-old mice (n = 7-8/group) using NMR (Echo MRI). Food intake, energy expenditure, and locomotor activity were monitored at 17-19 weeks of age using a combined indirect calorimetry system (TSE Systems) (n = 7-8/group). Mice were acclimated in the monitoring chambers for 2 days then data were collected for 3 days. These physiological measures were performed at the Rodent Metabolic Core of Children’s Hospital of Los Angeles. Glucose tolerance tests (GTTs) were conducted in 10- to 11-week-old mice (n = 9-11/group) through i.p. injection of glucose (1.5 mg/g body weight) after overnight fasting. Blood glucose levels were measured at 0, 15, 30, 45, 60, 90, 120, and 150 min post-injection. Serum leptin, insulin, T3, T4 levels were assayed in 18-21-week-old fed mice and corticosterone levels were assayed in 18-21-week-old fed mice (n = 8-11/group) using ELISA kits (Millipore, Calbiotech and Enzo Life Sciences, respectively).

##### Fluorescent *in situ* hybridization

Antisense digoxigenin-labeled riboprobes were generated from plasmids containing PCR fragments of *Pomc.* Briefly, sections were postfixed for 10 min in 4% paraformaldehyde. Then, sections were incubated with 1ug/ml Proteinase K (Promega) for 5 min at 37°C and 15 min at RT, respectively. They were then incubated for 10 min in 0.1 M triethanolamine (TEA), pH 8.0, and then for 5 min at room temperature in 100 mL 0.1 M triethanolamine (TEA) with 500 uL glacial acid acetic. Tissue was pre-hybridized for 2 h at 62°C in hybridization buffer (66% (v/v) deionized formamide, 13% (w/v) dextran sulfate, 260 mM NaCl, 1.3 X Denhardt’s Solution, 13 mM Tris pH 8.0, 1.3 mM EDTA pH 8.0). Probes were denatured at 80°C for 5 min before being added to the hybridization buffer, along with tRNA and DTT for a final concentration of 0.5mg/ml tRNA and 10mM DTT. Tissue was hybridized overnight at 62°C. After washes in stringency solutions, sections were blocked in TNB solution (0,5% blocking reagent, Roche). Tissue sections were then incubated for 1 hr at RT in a horseradish peroxidase-conjugated sheep anti-DIG antibody (1:400, Roche Applied Sciences). DIG was visualized using a TSA PLUS Biotin Kit (Perkin Elmer). After washes, sections were incubated for 30 min in a 1:50 dilution of the Biotin Amplification Reagent working solution (prepared following manufacturer’s instructions), before incubating for 1 hr in 1:200 streptavidin conjugated to cyanin 2 (Jackson Immunoresearch). Sections were counterstained using bisbenzamide (1:10,000, Invitrogen), to visualize cell nuclei, and coverslipped with buffered glycerol (pH 8.5).

##### Histomorphological assessment of white adipose tissue

Male mice were anesthetized at 16-17 weeks of age (n = 5/group). Epididymal adipose tissue was collected, fixed in a 4% paraformaldehyde solution, sectioned at 5 um, and then stained with a Perilipin A antibody (1:1,000, Sigma) using standard procedures. Images were taken with a Zeiss LSM 710 confocal microscope with a 20X objective. Determination of mean size (μm^2^) was measured using ImageJ software (NIH, ImageJ 1.39 T). The average adipocyte size measured from three sections in each mouse was used for statistical comparisons.

### Quantification and Statistical Analysis

All values were represented as the mean ± SEM. Studies in cellular models are from at least 3 independent experiments. Numbers for every experiment are found in the relevant part of the STAR Methods. Explant co-culture assays are derived from 4-28 explants in 3-6 independent experiments. Mice studies are from 3–11 animals per group. Statistical analyses were conducted using GraphPad Prism (version 6.07). Datasets with only two independent groups were analyzed for statistical significance using unpaired two-tailed Student’s t test. Datasets with more than two groups were analyzed using one-way analysis of variance (ANOVA) followed by the Bonferroni posthoc test. For statistical analyses of body weight and GTT (mice), we performed two-way ANOVAs followed by Bonferroni’s posthoc test. Statistically significant outliers were calculated using Grubb’s test for outliers. p ≤ 0.05 was considered statistically significant. Statistical significance is represented as ^∗^p < 0.05, ^∗∗^p < 0.01 and ^∗∗∗^p < 0.001.

## Supplemental Information

Table S1. Very Rare (MAF < 0.025%) Predicted Functional Variants Identified in the Gene-Set Enrichment Analysis of SCOOP (Severe Obesity) Cases vs. Controls from the INTERVAL Cohort, Related to Table 1 Annotation was performed using ANNOVAR with hg19 databases 'gnomadAD exome collection' (date 20170311), ExAC0.3 (date 20151129), avsnp147 (date 20160607) and ensGene (date 20151212), and the gnomAD browser (http://gnomad.broadinstitute.org/). gnomAD_exome_NFE (%) reports 100% x MAF (minor allele frequency) in Non-Finnish European (NFE) gnomAD exomes; "0%" indicates a variant detected in gnomAD exomes with MAF=0 in the NFE sample; “.” indicates variant not found in gnomAD exomes. Abbreviations: NA – not applicable; rsID – reference SNP cluster identifier. None of the SEMA3B transcripts in Genome reference consortium build 37 (*GRCh37*) are listed as producing proteins. Therefore, the gene-set enrichment analysis was performed on all genes, except for SEMA3B.
