# Supplementary material for: Human Semaphorin 3 Variants Link Melanocortin Circuit Development and Energy Balance
Source: Cell. 2019 Feb 7;176(4):729–742.e18. doi: 10.1016/j.cell.2018.12.009 (PMC6370916; doi:10.1016/j.cell.2018.12.009)
Supplement: Document S2. Tables S2 and S3 [file mmc2.pdf]

Table S1. Very rare predicted functional variants identified in the gene-set enrichment analysis of severe obesity cases vs healthy controls from the INTERVAL cohort. Related to Table 1.

See excel spreadsheet

Functional variants with a minor allele frequency (MAF) of <0.025% were considered very rare. Annotation was performed using ANNOVAR with hg19 databases 'gnomadAD exome collection' (date 20170311), ExAC0.3 (date 20151129), avsnp147 (date 20160607) and ensGene (date 20151212), and the gnomAD browser (http://gnomad.broadinstitute.org). gnomAD_exome_NFE (%) reports 100% x MAF (minor allele frequency) in Non-Finnish European (NFE) gnomAD exomes; "0%" indicates a variant detected in gnomAD exomes with MAF=0 in the NFE sample; "." indicates variant not found in gnomAD exomes. Abbreviations: NA – not applicable; rsID – reference SNP cluster identifier. None of the SEMA3B transcripts in Genome reference consortium build 37 (*GRCh37)* are listed as producing proteins. Therefore the gene-set enrichment analysis was performed on all genes except for SEMA3B.

Table S2. Enrichment analysis of very rare variants in severely obese cases and healthy controls from the INTERVAL cohort. Related to Table 1.

Types of gene level analysis	MAF	Genes≥1variant	case/ control allele count	OR	P value	Adjusted P value	P Bonferonni
missense/LoF	0.025%	12	43/134	1.404	8.55E-03	1.00E-02	**2.00E-02**
LoF only	0.025%	7	6/19	1.382	1.69E-01	1.99E-01	3.98E-01

Columns: types of gene-level analysis (for all missense or for predicted loss of function (lof) variants are shown with MAF=minor allele frequency threshold; number of genes with at least 1 variant, case/control allele count, OR=odds ratio, P-value (unadjusted), P value after empirical adjustment for testing for multiple genes, P Bonferroni – adjusted p-value after Bonferroni adjustment for multiple-test correction for 3 sets.

Table S3. Net effects of rare human variants in SEMA3s on function in cells. Related to Figure 1.

Gene	Variant	Secretion	Collapse corrected for secretion	Other effects where tested	Net effect
**SEMA3A**	**R350T**	↔	↔		WT-like
**SEMA3A**	**K600M**	↓	↔		↓ secretion
**SEMA3B**	**P296L**	↓	↑	↓ total neuronal and Pomc projections	↓ secretion
**SEMA3B**	**F355L**	↓	↑		↓ secretion
**SEMA3C**	**R739Q**	↑	↓	↔ Pomc projections	↓ collapse
**SEMA3D**	**Y199S**	↔	↓		↓ collapse
**SEMA3D**	**R265H**	↑	↓		↓ collapse
**SEMA3D**	**D380H**	↔	↓		↓ collapse
**SEMA3D**	**T397A**	↔	↓	↓ neuronal (but not Pomc) projections	↓ collapse
**SEMA3D**	**N444S**	↔	↔		WT-like
**SEMA3D**	**D640Y**	↔	↔	↑ Pomc projections	↑Pomc projections
**SEMA3D**	**R773G**	↑	↓		↓ collapse
**SEMA3E**	**R167G**	↑	↓		↓ collapse
**SEMA3E**	**K711N**	↑	↔		↑ secretion
**SEMA3F**	**E88K**	↓	↔		↓ secretion
**SEMA3G**	**A86S**	↑	↔		↑ secretion
**SEMA3G**	**E478D**	↓	↔	↓ Pomc projections	↓ secretion
**SEMA3G**	**R561W**	↔	↔		WT-like
**SEMA3G**	**R728C**	↓	↔	↓ dimerisation	↓ secretion

The results of functional assays on the secretion of, and signaling by, mutant forms of Semaphorin 3s are summarised. To estimate the net effect of each mutant, we have assumed that the total amount of ligand secreted may have a greater impact on signaling than the amount of cell collapse induced by each mutant (after adjusting for the amount secreted). Statistically significant differences for each mutant in each assay when compared to WT are shown: ↑ = increase; ↓= decrease; ↔ = no difference.
